# A Review of Spatter in Laser Powder Bed Fusion Additive Manufacturing: In Situ Detection, Generation, Effects, and Countermeasures

**DOI:** 10.3390/mi13081366

**Published:** 2022-08-22

**Authors:** Zheng Li, Hao Li, Jie Yin, Yan Li, Zhenguo Nie, Xiangyou Li, Deyong You, Kai Guan, Wei Duan, Longchao Cao, Dengzhi Wang, Linda Ke, Yang Liu, Ping Zhao, Lin Wang, Kunpeng Zhu, Zhengwen Zhang, Liang Gao, Liang Hao

**Affiliations:** 1 Gemological Institute, China University of Geosciences, Wuhan 430074, China; 2 Department of Mechanical Engineering, Tsinghua University, Beijing 100084, China; 3 Wuhan National Laboratory for Optoelectronics, Huazhong University of Science and Technology, Wuhan 430074, China; 4 Diligine Photonics Co., Ltd., Guangzhou 510000, China; 5 TSC Laser Technology Development (Beijing) Co., Ltd., Beijing 100076, China; 6 School of Machinery and Automation, Wuhan University of Science and Technology, Wuhan 430081, China; 7 School of Aerospace Engineering, Huazhong University of Science & Technology, Wuhan 430074, China; 8 Shanghai Engineering Technology Research Center of Near-Net-Shape Forming for Metallic Materials, Shanghai Spaceflight Precision Machinery Institute, Shanghai 201600, China; 9 Faculty of Mechanical Engineering & Mechanics, Ningbo University, Ningbo 315211, China; 10 Department of Microtechnology and Nanoscience, Chalmers University of Technology, 41296 Gothenburg, Sweden; 11 Nanjing Chamlion Laser Technology Co., Ltd., Nanjing 210039, China; 12 The State Key Laboratory of Mechanical Transmissions, Chongqing University, Chongqing 400044, China; 13 State Key Laboratory of Digital Manufacturing Equipment and Technology, School of Mechanical Science and Engineering, Huazhong University of Science and Technology, Wuhan 430074, China

**Keywords:** spatter, laser powder bed fusion, in situ detection, generation mechanism, detrimental effects, counter-measures, additive manufacturing

## Abstract

Spatter is an inherent, unpreventable, and undesired phenomenon in laser powder bed fusion (L-PBF) additive manufacturing. Spatter behavior has an intrinsic correlation with the forming quality in L-PBF because it leads to metallurgical defects and the degradation of mechanical properties. This impact becomes more severe in the fabrication of large-sized parts during the multi-laser L-PBF process. Therefore, investigations of spatter generation and countermeasures have become more urgent. Although much research has provided insights into the melt pool, microstructure, and mechanical property, reviews of spatter in L-PBF are still limited. This work reviews the literature on the in situ detection, generation, effects, and countermeasures of spatter in L-PBF. It is expected to pave the way towards a novel generation of highly efficient and intelligent L-PBF systems.

## 1. Introduction

Additive manufacturing (AM) is widely used in aerospace, medicine, jewelry, and other industries because of its rapid fabrication [1,2], low cost, and the ability to print parts with complex geometries [3,4]. Today, many developed and developing countries regard AM technology as a fifth industrial revolution and make many efforts in the development of AM. The United States Department of Defense (DoD) released the Department of Defense Additive Manufacturing Strategy [5] to stimulate the development of AM applications in national defense. Meanwhile, the Office of the Under Secretary of Defense released the first policy paper, DoD 5000.93 Directive Use of Additive Manufacturing in the Department of Defense [6], which promoted the implementation of the AM strategy. The Ministry of Science and Technology of the People’s Republic of China released the 2022 annual project application guide for the key projects of additive manufacturing and laser manufacturing under the 14th Five-Year National Key R&D Program [7] to establish a new standard system for AM that is consistent with international standards. Additionally, AM and laser manufacturing are two of the important tasks of the National Program for Medium-to-Long-Term Scientific and Technological Development and Made in China 2025. The EU began funding projects on AM technology as early as the first Framework Program for Research and Technological Development. Under these conditions, AM technology has advanced significantly and rapidly in developing standard systems, key technologies, and multi-industry applications.

AM technology emerged in the 1990s and has been under development for approximately three decades [8]. Unlike “subtractive manufacturing” (e.g., cutting, drilling, and milling) and “equal-material manufacturing” (e.g., welding, casting, and forging), AM is built on 3D models [9], relies on layer by layer printing-extrusion, sintering [10,11], melting, light curing, and jetting to form solids from metallic or non-metallic materials [12,13].

**Figure 1:**
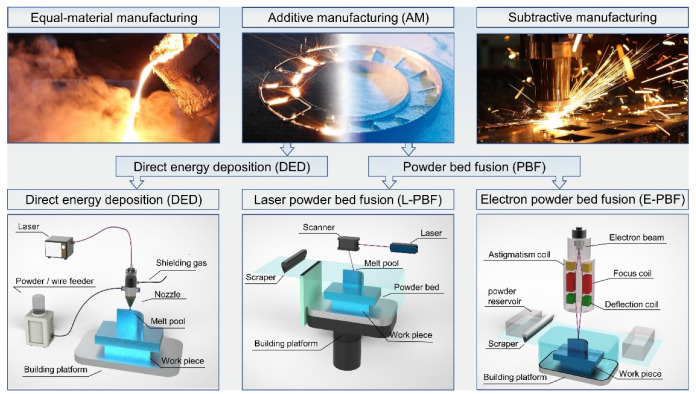
Classification of metal manufacturing processes: equal-material manufacturing, additive manufacturing [22], subtractive manufacturing.

Metal AM is one of the most difficult and cutting-edge AM technologies. As shown in Figure 1, metal AM technologies can be divided into two categories, direct energy deposition (DED) and powder bed fusion (PBF) [14,15]. PBF is one of the AM technologies used to fabricate metal objects from powder feedstocks with two kinds of input energy: laser and electron [16,17,18]. In the printing process, the metal powder bed is melted by the high energy source with a designed pattern using a layer by layer printing strategy [19,20,21].

Figure 1 also illustrates the forming principle of laser powder bed fusion (L-PBF), which is widely used today to rapidly manufacture parts with complicated shapes, a fine grain size, high densities, and superior mechanical properties [23,24]. Although it can currently fabricate complicated metal parts [25,26], the reliability and stability of the printing process remain inadequate [27]. There are still defects in L-PBF processing that decrease the density and affect the mechanical characteristics of the part or even result in fabrication failure. The many unresolved problems with L-PBF become a barrier to the expansion of L-PBF applications.Spatter is generated in conventional laser welding and cutting, DED, and L-PBF. Spatters are the particles ejected from a melt pool during the laser–metal interaction [28]. In conventional laser welding and cutting, the laser scanning path is relatively simple, with few overlap regions between the scanning paths, and DED has a lower scanning velocity and a larger spot than L-PBF. However, L-PBF is a powder-bed-based technology, and the printing process is more complicated than that of the three technologies mentioned above, which results in a more complex spatter behavior. Furthermore, during multi-laser L-PBF, the thermal and stress cycling, melt pool characteristics, spatter behavior, and metal vapor evolution will be definitely different from that of the single-laser PBF. The detection of spatter under multi-laser L-PBF is more difficult.

For this reason, studies on L-PBF spatter are becoming very urgent. Spatter as a by-product of L-PBF is unpreventable [29,30]. It is a detriment to the forming process, and the part and the redeposited spatters can destroy the original well-built powder layer, resulting in non-fusion defects [31,32]. Due to the uniqueness of L-PBF, the undesired effects of spatter are amplified during the layer-by-layer process. Spatter affects the subsequent re-coating and melting of the powder, resulting in internal defects in the produced part or the part failing to form.

As spatter has a significant effect on L-PBF, it can be used to represent the L-PBF machining state. Spatter contains a plethora of information and can be used in various ways to analyze the manufacturing processing of L-PBF. By observing and quantifying the spatter, it is possible to establish an intrinsic correlation between spatter and the part quality, enabling a more comprehensive understanding of the L-PBF process to solve the problems of insufficient stability and reliability, allowing this technology to be popularized and applied more widely.

**Figure 2:**
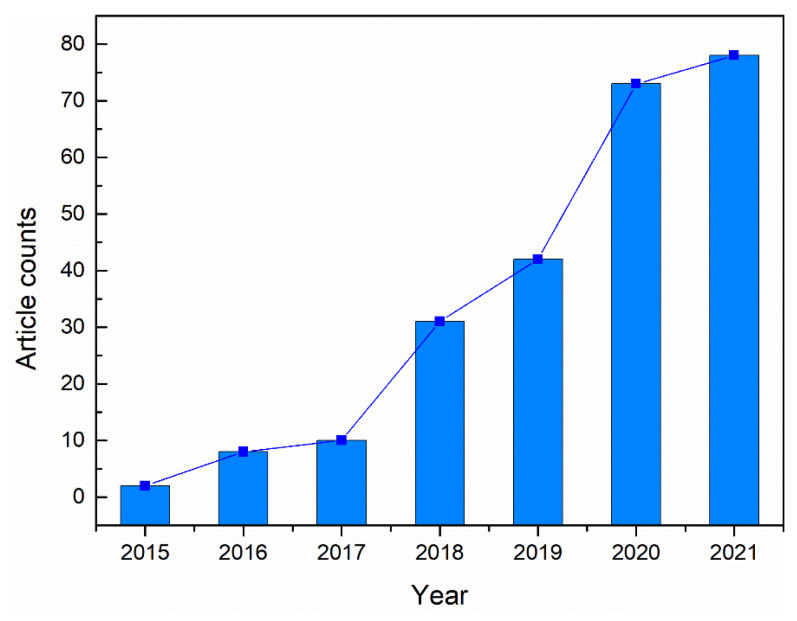
Number of articles about L-PBF spattering since 2016 (Topic: [“laser-powder bed fusion” and “spatter”] or [“selective laser melting” and “spatter”]) Database: Web of Science.

Recently, the research concerning the spatter during L-PBF has received more and more extensive attention. In this work, we review academic publications concerning L-PBF spatter in the Web of Science database from 2015 to date (Topic: [“laser-powder bed fusion” and “spatter”] or [“selective laser melting” and “spatter”]). Figure 2 shows the trend in the number of articles on this topic over the last several years.

This article builds on previous research by reviewing a synthesis of in situ spatter detection systems, spatter detection equipment, the generation of spatter and its associated disadvantages, and current approaches for the suppression and removal of spatter. Finally, the future of research on L-PBF spatter is discussed.

## 2. Laser Powder Bed Fusion Spatter In Situ Detection Device

The L-PBF detection system can be categorized as: static detection (imaging of spreading powder and deformation) and dynamic detection (characterization of melt pool, spatter, and vapor plume).

The spatter generated by conventional laser welding, cutting, and DED is similar to that produced by L-PBF and is caused by the interaction between the laser and the metal material. However, L-PBF has a smaller spot (~10^1^ to 10^2^ µm), a smaller melt pool (up to 100 µm), a shorter lifetime (~10 ms), and a higher scanning velocity (~10^2^ to 10^3^ mm/s) compared to laser welding, cutting, and DED [33]. Furthermore, in L-PBF, the laser interacts with the powder bed and the metal part more than once, resulting in a greater number and variety of spatters and complicating in situ spatter detection.

The laser–powder bed interaction produces the melt pool, spatter, and vapor plume (even plasma). The trajectory of the melt pool is in the plane of the laser path and can be predicted according to the strategy path, whereas the motion of the spatter is in a 3D space, and its trajectory is complex and difficult to predict. So, the detection of spatter is more difficult. Spatter can be divided into hot droplet spatter (mainly from the instability of the melt pool) and cold powder spatter (mainly driven by the vapor-induced entrainment of the protective gas). Both of them can be detected with the visible-light camera equipped with an illumination source, and the relevant collected information can be used to analyze them.

**Figure 3:**
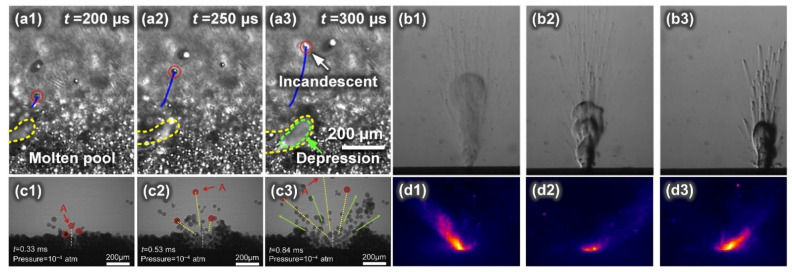
Characteristics obtained from different in situ detection techniques: (**a1**–**a3**) time series snapshots taken by visible light high-speed camera (Reprinted with permission from Ref. [34]. Copyright 2019 Elsevier B.V.); (**b1**–**b3**) high-speed schlieren images during single track scans (Reprinted with permission from Ref. [35]. Copyright 2018 Springer Nature.); (**c1**–**c3**) dynamic X-ray images showing powder motion, A is the ejected powder (Reprinted with permission from Ref. [36]. Copyright 2018 Elsevier B.V.); (**d1**–**d3**) three consecutive frames of an infrared video acquired during L-PBF (Reprinted with permission from Ref. [37]. Copyright 2018 Elsevier B.V.).

According to various studies, the following methods are currently available for L-PBF spatter detection: (1) a visible-light high-speed camera, (2) X-ray video imaging, (3) infrared video imaging, and (4) schlieren video imaging. These detection techniques can detect different characteristics, as shown in Figure 3 and Table 1.

### 2.1. Visible-Light High-Speed Detector

**Figure 4:**
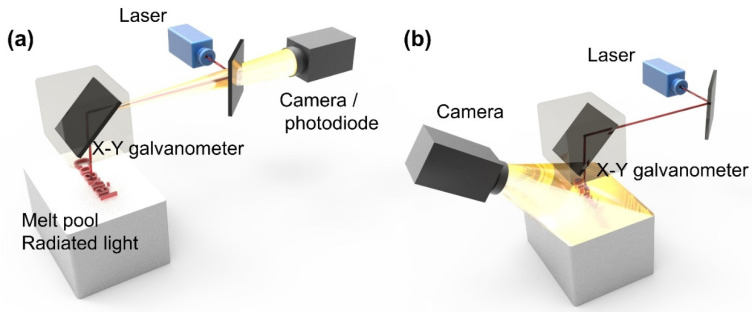
L-PBF in situ detection system: (**a**) coaxial (sharing the optical path with the laser); (**b**) off-axial (at a slight angle to the laser optical path).

There are two main methods for observing L-PBF with a high-speed visible-light camera: coaxial and off-axis. In Figure 4a, the camera shares the same optical path with the laser in a coaxial solution. In Figure 4b, the camera is placed at an angle to the optical path of the laser for viewing in Figure 4a, an off-axis solution.

Coaxial in situ detection of a commercial L-PBF machine requires extensive modification of the machine, and it is still difficult to obtain clear images because of the distance between the optical path system and the powder bed in the L-PBF machine. Another hindrance is the small optical aperture of the scanner and F-theta lens, which results in low magnification. In addition, the low reflectivity of the scanner and the low transmittance of the F-theta lens also reduce the temporal and spatial resolution of imaging, and these two characters are vital for the analyzing the trajectory and behavior of spatters. To overcome the disadvantages of coaxial in situ detection, Zhang et al. [38] improved the optical path, built segmentation algorithms, and demonstrated the algorithms’ efficiency in dealing with defocused and distorted spatter images.

**Figure 5:**
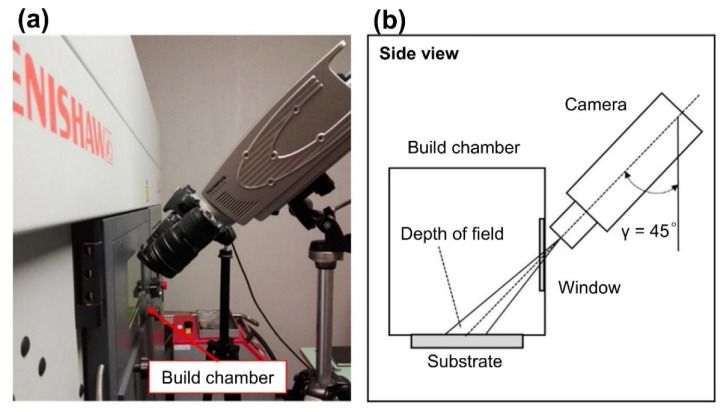
Off-axial detection system: (**a**) off-axial mounting of the high-speed camera outside the build chamber; (**b**) off-axial high-speed camera is at an angle of 45° to the plane of the observed powder bed. (Reprinted with permission from Ref. [39]. Copyright 2017 Elsevier B.V.).

Unlike the coaxial solution, the off-axis solution, which places the detection device at an angle to the powder bed, enables spatter detection without altering the existing L-PBF equipment, as shown in Figure 5. The system is more adaptable and simpler to alter, and because the detection system does not share the optical path of the laser, it is not constrained by the laser’s original optical path and can be used to detect spatter at higher magnification and frame rates than those of the coaxial system. Due to these factors, off-axial in situ detection system is becoming increasingly popular.

In the case of coaxial detection, the detection equipment and external light source affect the final detection findings. Yang et al. installed a high-speed camera (pco. Dimax HS4, 3000 fps) outside the L-PBF machine at a 65° angle to the working platform to detect the spatter. Due to the little difference in brightness between the powder spatter and power bed within the view field of the high-speed camera, only the droplet spatters were detected, but not the nonmolten powder particles. Tan et al. [40] used a computational technique to analyze the obtained images, segmenting each block to extract the spatter. In the same year, Yin et al. [41] introduced an external light source (a CAVILUX^®^ pulsed high-power diode laser light source) and a high-speed camera (Phantom V2012) to detect the spatter and obtain clearer images. After that, this in situ detection system was used to investigate the correlation between ex situ melt track properties and in situ high-speed, high-resolution characterizations [34].

**Figure 6:**
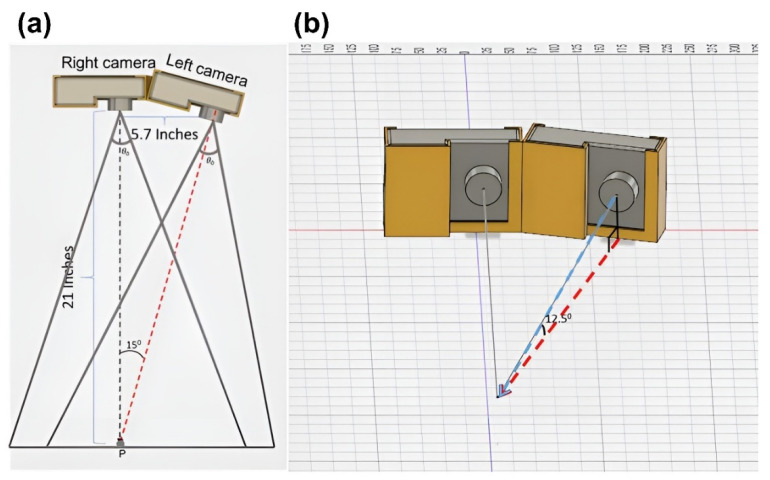
Spatter detection system for stereo vision: (**a**) the horizontal angle between the two sensors is 15°; (**b**) the vertical direction of the sensor is 12.5° from the observation object. (Reprinted with permission from Ref. [43]. Copyright 2018 University of Texas at Austin.).

The above studies were based on the monocular camera, and the picture information collected was in a 2D space. By combining multiple cameras and using image processing arithmetic, 3D information of spatter and its mobility can be gathered. Based on the use of monocular sensors, Luo et al. [42] innovatively proposed the use of acoustic signals combined with deep learning for spatter detection, demonstrating the feasibility of the acoustic signal detection of spatter behavior. Due to the dimensional limitation of the 2D image (acquired by the monocular sensor), it is difficult to accurately calculate the behavioral information of the spatter and obtain accurate spatter trajectory, velocity, and other information. A binocular stereo detector can obtain the spatter information in two viewing angles. By using the multi-directional information, its algorithm can present the 3D trajectory and velocity of a single spatter, and the obtained information is more accurate than those of a monocular sensor. Barret et al. [43] established a stereo vision spatter detection system for spatter tracking analysis at a cost of less than USD 1000 using two slow-motion cameras (FPS1000 by The Slow Motion Camera Company), as illustrated in Figure 6. Later, Eschner et al. [44] combined two ultra-high-speed cameras with algorithms to create a 3D tracking system for measuring spatter in L-PBF. Visible in situ detection systems for L-PBF in recent years are summarized in Table 2.

### 2.2. Invisible-Light In Situ Detection

For the invisible-light in situ detection of L-PBF, the imaging technologies mainly include X-ray imaging, schlieren video imaging, infrared imaging, and thermal imaging.

**Figure 7:**
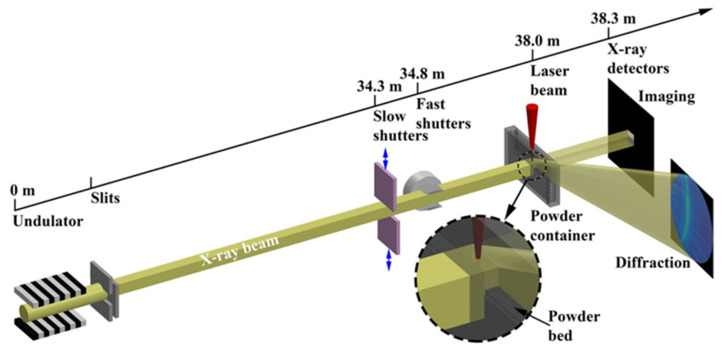
Schematic of the high-speed X-ray imaging and diffraction experiments on laser powder bed fusion process at the 32-ID-B beamline of the Advanced Photon Source. A pseudo-pink beam with a first harmonic energy of 24.4 keV (λ = 0.508Å) is generated by a short-period undulator. The laser irradiates the micro-powder bed sample from the top, the X-rays penetrate from the side of the sample. The imaging and diffraction detectors are placed approximately 300 mm downstream from the sample. The inset surrounded by the dashed circle enlarges the view of the laser-sample and X-ray-sample interaction. The distance of each component from the source is labeled on top. (Reprinted with permission from Ref. [28]. Copyright 2017 Springer Nature.).

X-rays have a short wavelength, high energy, and high penetration ability. High-strength X-rays can penetrate a certain thickness of metal with high temporal and spatial resolution, which is the preferred method for many L-PBF spatter studies [35]. A schematic diagram of an X-ray system is shown in Figure 7. As one of the most productive X-ray sources globally, the Advanced Photon Source (APS) in the Argonne National Laboratory provides experimental conditions for many researchers. More than 5500 researchers per year use X-rays produced by APS to do experiments. Many of those researchers use those X-rays to detect L-PBF spatter. For example, Zhao et al. [28] pioneered the use of high-speed X-rays (harmonic energy 24.4 keV) for in situ characterizations of L-PBF progress. Guo et al. [36] found transient spatter dynamics in L-PBF using a high-speed, high-resolution, and high-energy X-ray imaging technique. Ross Cunningham et al. quantified the keyhole in Ti-6Al-4V powder during laser melting based on X-ray image information [55]. Leung et al. raised the X-ray power (monochromatic X-ray power: 55 keV) and studied stainless steel (316L) and 13–93 bioactive glass. They found that melt pool wetting and vapor-driven powder entrainment are key track growth mechanisms for L-PBF [57]. A summary of X-ray in situ detection is shown in Table 2.

Due to the Schlieren video imaging and infrared imaging to picture previously invisible light or materials, these two technologies are also used for the in situ detection of spatter. Schlieren video imaging, used to detect the plume in L-PBF, can visualize the invisible substance by measuring its refractive index. Bidare et al. [58] used a combination of a high-speed camera and schlieren video imaging to capture images of the denuded region, laser plume, and argon atmosphere, and explained the relation between the powder-bed denuded region and spatter. An infrared camera can collect the light emitted by an infrared light source. Ye et al. [53] used infrared cameras to detect the properties of the original plume and spatter. Grasso et al. used the plume as the information source and examined it with an infrared camera to rapidly discover processing defects and unstable states [59,60].

### 2.3. Data Processing during Spatter Detection

Spatter image obtained from in situ detection requires post-processing to enable the extraction and analysis of spatter behaviors.

#### 2.3.1. Spatter 2D Image Processing Algorithm

Algorithms for 2D image processing are less complex than those for 3D image processing. Tan et al. [40] captured spatter images using Kalman filter tracking, segmented the images with grayscale and edge information, and obtained spatter information using fully convolutional networks and Mask R-CNN. Yin et al. [61] projected the 3D spatter trajectory into a 2D plane with image processing, used a filtering technique to improve the sharpness of the spatter image, and tracked the spatter motion information frame by frame using ImageJ.

#### 2.3.2. Spatter 3D Image Processing Algorithm

Barrett et al. used a low-cost binocular sensor for spatter detection, laying a foundation for future analysis of the data [43]. Eschner et al. [44] used algorithms in a binocular sensor system to carry out many processes on the images, including (1) identifying particle positions and calibrating the camera system, (2) matching particles between multi-camera images, (3) determining the 3D coordinates, (4) using a priori knowledge of processes and particles to distinguish ghost particles from real particles, (5) tracking particles, and (6) processing the 3D data. Those processes require more complex algorithms to complete. Currently, they have enabled the construction of a quadruple-eye sensor system, which uses a third camera to achieve the recognition of ghost particles. However, relative to the binocular sensor detection system, the quadruple-eye sensor detection system must process a larger amount of information that is more difficult to process [45].

### 2.4. Full-Cycle Detection of Spatter in L-PBF

During L-PBF process, the full cycle of the spatter can be divided into three stages: the initial stage (generation), the flight stage (ejection), and the fall-back stage (re-deposition). The detection of the spatter in these three stages is conductive to the deep understanding of the origin of the spatter, the correlation of the spatter and defect, and the influence of the spatter on the part.

- **Initial stage (generation, adjacent to the melt pool):** The positions of the generation of both the cold spatters and hot spatters are adjacent to the melt pool. The ultra-high-frame-rate in situ detection using a high-temporal-spatial-resolution off-axis camera combined with the illumination light source can obtain a clear morphology of spatters, which helps to reveal the mechanism of the spatter generation.
- **Flight stage (ejection, away from the powder bed):** The amount of spatter and the ejection angle significantly affect the internal defect of the part. The spatter trajectory, ejection velocity, ejection angle, and spatter size of the spatter should be obtained to investigate the intrinsic correlation between the spatter and the defect. A long monitoring time, high-frame-rate in situ detection system, along with the laser path using multi-sensors, is applied to capture the spatter flight (even with 3D information). The high-throughput data during L-PBF process can be used for the statistics analysis of spatter characterization. In general, only hot spatters are detected in this stage to reduce the processing pressure of the monitoring system.
- **Fall-back stage (re-deposition, close to the powder bed):** The spatter eventually redeposits on the powder bed and parts, which affect re-coating and part quality. A layer-by-layer in situ detection with a wide field-of-view and high-spatial-resolution camera can obtain high quality images of the powder and parts. The image data employing algorithms extract and confirm the size and location of the redeposited spatter, which helps in predicting the forming quality of the parts and the location of the defect.

### 2.5. Differences In Situ Detection between Spatter and Melt Pool 

Due to the complexity of spatters, algorithmic requirements are higher than for melt pool detection. Generally speaking, the melt pool goes along with the laser spot and the melt pool movement is in the 2D trajectory, but the spatter movement is in the 3D trajectory, so the detection of the spatter must be extended to 3D, which requires more in situ sensors and more information needs to be processed.

(1) Compared with the detection of the melt pool, the spatter, with a micro size and extensive range of motion in the 3D space, is much more difficult to be detected, which requires multiple sensors, up to four sensors, with micron spatial resolution.
(2) Additionally, the melt pool is generated by the action of the laser in the metal powder bed, and its trajectory can be predicted according to the pre-defined laser path. In contrast, the trajectory of spatter is hard to be predicted due to the high-speed random motion in the 3D space, which requires sensors with a higher temporal resolution up to microseconds to detect the whole process of motion trajectory deflection.
(3) The data of spatter collected using sensors with high spatial resolution and high temporal resolution are several orders larger than the data of melt pool detection. Therefore, the data processing of spatter detection is more complex, which puts higher demands on the algorithm.

As a result, the observation of the spatter and data processing, is much more challenging than the detection of the melt pool. The complexity of the spatter detection algorithms is further increased by 3D detection systems with multiple sensors.

## 3. Mechanism of Spatter Generation

Under the interaction with a high-energy laser in L-PBF, metal powders are melted to form a melt pool when the temperature attains the melting point, then vaporized to form metal vapor or even a plasma plume when the surface temperature of the melt pool surpasses the boiling point. The different phases (solid, liquid, and vapor) significantly interact with each other during L-PBF process, among which the vapor–solid interaction and vapor–liquid interaction are the main mechanism of spatter generation. Therefore, it is necessary to investigate the mechanism of spatter generation.

### 3.1. Spatter Classification

**Figure 8:**
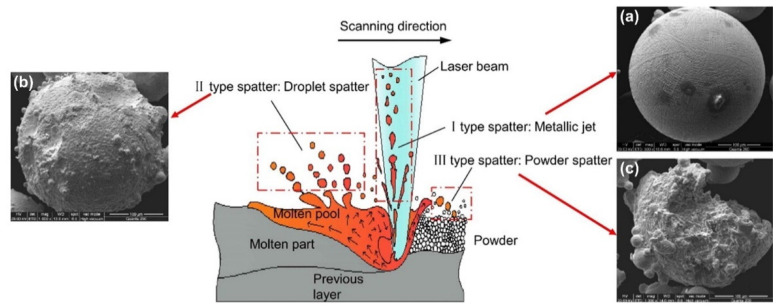
Formation mechanisms of different types of spatter during L-PBF: (**a**) morphology of spherical spatter (Type-I spatter); (**b**) morphology of coarse spherical morphology (Type-II spatter); (**c**) morphology of irregular spatter (Type-III spatter). (Reprinted with permission from Ref. [63]. Copyright 2016 Elsevier B.V.).

The spatters generated in L-PBF are in a different morphology, and a variety of parameters affect spatter generation. Until now, there has been no common definition of spatter categorization. Liu et al. [62] performed L-PBF single-pass scanning experiments with 316L stainless steel powder, reflecting the dynamic behavior of spatter perpendicular to the single-track scanning direction by the high-speed imaging technology. They divided the spatter into two categories: droplet spatter and powder spatter. It is known that the spatter formation mechanism can be demonstrated as the hot spatter ejection, mainly driven by the instability of the melt pool due to the vapor-induced recoil pressure, and the cold spatter ejection, mainly driven by the vapor-induced entrainment of the protective gas. Wang et al. [63] used a high-speed camera to record the dynamic spattering process of Co–Cr alloys during L-PBF manufacturing and investigated the spatter generation mechanism in further detail. As shown in Figure 8, they recognized three major sources of spattering: recoil pressure, the Marangoni effect, and the heat effect in the melt pool. These three different sources of spattering led to three types of spattering morphologies.

According to Ref. [63], there are three types of spatters: (ⅰ) The Type I spatters are associated with the extreme expansion of the gas phase. The spontaneous metal liquid flowing will occur from the high-temperature bottom of the excavation to the low-temperature sidewall and edge at the back under the Marangoni effect. (ⅱ) Then, in this process, the recoil pressure can induce the jet of low-viscosity metal liquid, and this jetted liquid metal will divide into small drops in the flight process to minimize the surface tension; therefore, the Type II spatter is formed. (ⅲ) In the printing process, some metal liquid accumulates near the spot laser, and it can be easily squeezed by the blast wave and then interrupt these non-melted particles in the front-end area; then the Type III spatter will occur at the front of the melt pool.

**Figure 9:**
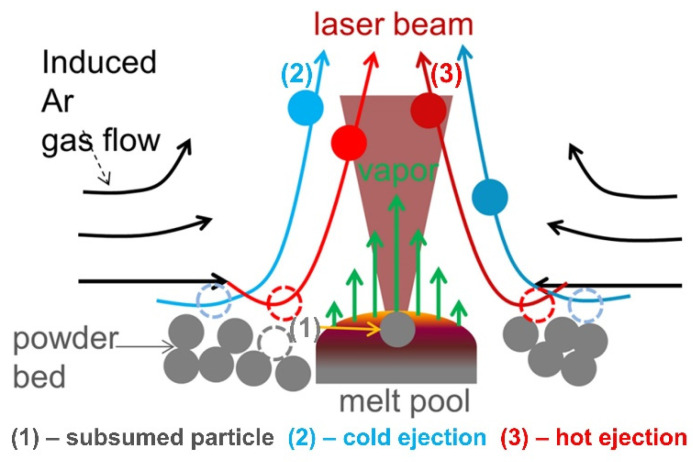
Schematic representation of the entrainment effect of metal-vapor-driven airflow on fine particles in the presence of a fixed laser beam. A vapor jet creates a zone of low pressure, which results in three different trajectories of entrained particles: (1) Particles with low vertical momentum are swept into the melt pool; (2) Particles with higher vertical momentum but originating > 2 melt pool widths away are swept into the trailing portion of the vapor jet, and ejected as cold particles; (3) particles with roughly the same vertical momentum as (2) but originating closer to the point of laser irradiation (<2 melt pool widths) are swept into or near the laser beam itself rapidly heat, and are ejected as incandescent, hot particles. (Reprinted with permission from Ref. [64]. Copyright 2017 Springer Nature.).

Ly et al. [64] used a high-speed camera to explore the influence of gas flow entrainment on spatter during L-PBF. They described the entrainment phenomena of 316L stainless steel powder and Ti-6Al-4V powder layers and divided spatter into three categories. As shown in Figure 9, 60% of the ejection was due to hot entrainment ejection at velocities ranging from 6 m/s to 20 m/s: 25% was cold entrainment ejection, which occurred at a velocity of 2 m/s to 4 m/s, and 15% was droplet breakup ejection from the melt pool as a result of the recoil pressure applied at a velocity of 3 to 8 m/s. Raza et al. [65] also found that spatter from the melt pool was less than that due to vapor-induced entrainment.

Young et al. [56] showed the characteristics and generation mechanisms of five unique types of spatter during L-PBF by in situ high-speed, high-energy X-ray video imaging: solid spatter, metallic ejected spatter, agglomeration spatter, entrainment melting spatter, and defect-induced spatter. They quantified the speed, size, and direction of metallic ejected spatter, powder agglomeration spatter, and entrainment melting spatter. The results showed that the metallic ejected spatter speed was the highest, and the size of the powder agglomeration spatter was the largest. The spatter direction was highly dependent on the characteristics of the depression zone, which was impacted directly by the metal vapor recoil pressure.

Whereas the above researches had classified spatter using an in-process analysis, the following is a study that classified spatter using a post-mortem analysis. Gasper et al. [66] divided the spatter into seven categories according to the size, morphology, and other descriptors, such as oxides and agglomeration derived from SEM analysis, namely: (1) particles similar to virgin gas-atomized particles, (2) particles morphologically different from those gas-atomized, (3) larger singular particles with different morphologies, (4) particles with oxide spots, (5) particles covered with oxide, (6) small particles, and (7) agglomerates. Yang et al. [67] studied the influence of the L-PBF parameters on the pore characteristics and mechanical properties of Al-Si10-Mg parts. Three distinct types of solidified droplets were detected: hollow droplets, semi-hollow droplets, and solid droplets. Hollow droplets and semi-hollow droplets were a major source of pores inside the sample. Table 3 summarizes current studies on the categorization of spatter generation during L-PBF.

### 3.2. Study of Droplet Spatter Ejected from “Liquid Base” of Melt Pool

The melt pool is a critical feature of L-PBF. Numerous studies on the spattering from the melt pool have been done using a numerical simulation, which avoided the high cost and inefficiency of repeated experiments. Khairallah et al. [68] studied the mechanism of spatter generation at the powder scale using a 3D high-precision model. The metal vapor exerted pressure on the melt pool during L-PBF, causing the emission of liquid metal. When the liquid metal was stretched, the column grew thinner and decomposed into tiny droplets because the surface tension tended to minimize the surface energy. Additionally, it was discovered that at the start of the scanning, it was rather easy to generate large-sized back-ejected spatters [69]. They assumed that the laser scanning velocity could not be kept constant at the beginning and end of the trajectory due to inertia, resulting in a deposition of a nonuniform energy density and causing such spatters. They proposed a stability criterion to eliminate back-ejected spatter effectively. Altmeppen et al. [70] proposed a method to simulate time-dependent particles and heat ejection from the moving melt pool. This model can predict the direction and velocity of spatter emission and determine the size and temperature of a single particle by evaluating the direction and velocity of local laser scanning.

**Figure 10:**
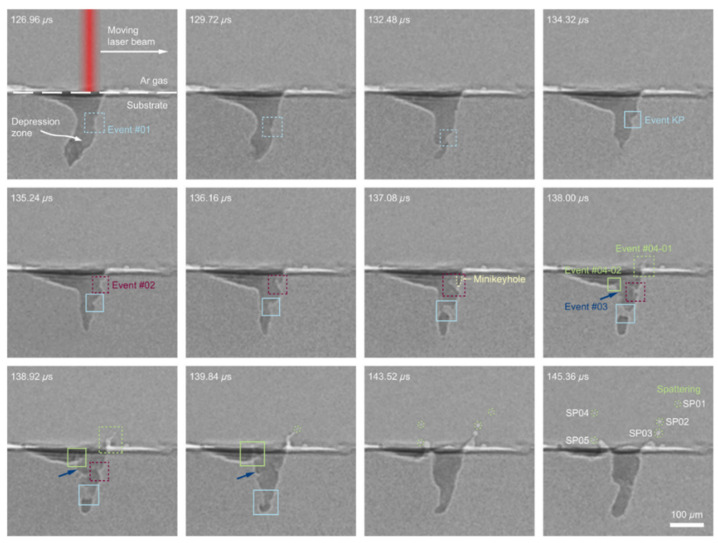
MHz X-ray images of metal spattering of Ti-6Al-4V during laser processing. **Event No. 01 (sky blue dashed rectangles):** A protrusion forms at the top surface and runs down along the front keyhole wall, accompanied by the keyhole morphology changing from a J-like shape to a reverse-triangle-like shape. **Event No. 02 (purple dashed rectangles):** A following protrusion appears, grows, and collapses around the horizontal center of the keyhole. A mini keyhole on top of the protrusion is outlined by a light yellow dashed curve. **Event No. 03 (dark blue arrows):** The local curvature on the rear keyhole wall changes. **Event No. 04 (light green dashed and solid rectangles):** Melt ligaments form, elongate, and break up into spatters (light green dashed circles numbered SP01–SP05). **Event KP (sky blue solid rectangles):** describes the formation and vanishing of a keyhole pore (Reprinted with permission from Ref. [71]. Copyright 2019 APS Physics).

In order to verify the intrinsic mechanism of the spatter generation, experiments were applied to detect the spatter using X-ray imaging and high-speed imaging. The explosion caused by the instability of the front wall of the keyhole, which resulted from the vaporization of the L-PBF volatile element, induced much droplet spatter. Zhao et al. used X-ray imaging to study the spatter behavior of Ti-6Al-4V powder during L-PBF. As illustrated in Figure 10, they demonstrated how the bulk-explosion induced by the instability of the front wall of the keyhole in the melt pool resulted in a considerable amount of droplet spatter [71]. Using in situ high-speed high-resolution imaging and thermodynamic analysis, Yin et al. investigated the vaporization and explosion behavior of alloy components in a Cu-10Zn alloy during L-PBF [72]. It was found that the explosion caused by the violent vaporization of a low boiling point also induced much droplet spatter and defects in the melt track.

**Figure 11:**
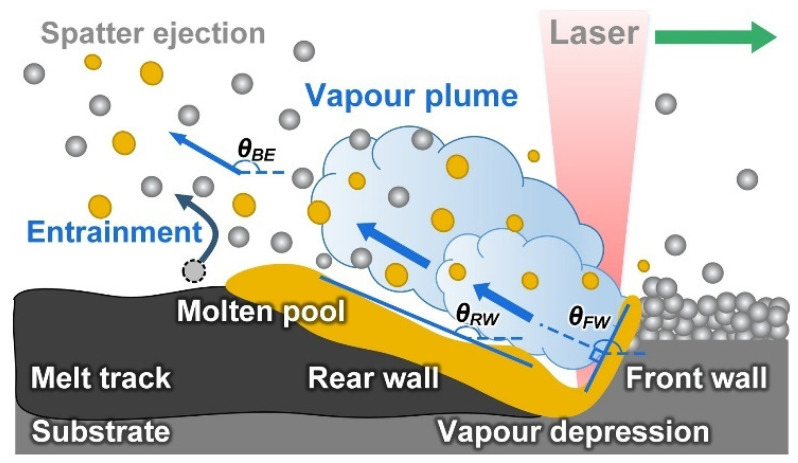
Schematic of the correlation between the depression zone in melt pool and the backward-ejected spatter during L-PBF. The inclined angles for the normal direction of the front depression wall *θ_FW_* and the inclination angle of the rear depression wall *θ_RW_* had the same trends with the average angle of the backward-ejected spatter (*θ_BE_*). (Reprinted with permission from Ref. [34]. Copyright 2019 Elsevier B.V.).

Using high-speed and high-resolution imaging technologies, Yin et al. [41] investigated the spatter behavior of Inconel 718 powder during L-PBF. The subthreshold ejection phenomenon was detected in which droplets emitted from the droplet column fell back to the melt pool. Later, the authors also studied the correlation between the ex situ melt track characteristics and the in situ high-speed and high-resolution characterization. They showed that the protrusion of the head of the melt trajectory was caused by the combined action of the backward flowing melt and the droplet ejection behavior in the melt pool [34]. Moreover, as illustrated in Figure 11, the melt pool first forms a depression under the action of the recoil pressure of the vapor; a high-energy laser beam impinges on the front wall of the depression, causing the surface of the front wall to quickly vaporize and generate a metal vapor that is perpendicular to this surface; the metal vapor expands and impacts the rear wall of the depression; finally, the spatter is formed and ejected backwards. The vertical metal vapor plume was identified as the principal reason for the melt pool spattering. Through in situ measurements of a typical forward spatter ejection angle, the vapor recoil pressure (approximately 0.46 atm) was quantified.

The development of various advanced in situ characterization methods provides new directions for spatter research. Wang et al. [48] used a high-speed camera to investigate the characteristics of the droplet spatter of 316L stainless steel powder during L-PBF process. Gould et al. [73] reported an in situ method to analyze the L-PBF process of Ti-6Al-4V and W powders by using high-speed X-ray and high-speed infrared imaging simultaneously. Combining both imaging of high-speed X-rays and high-speed infrared imaging, various phenomena can be identified including 3D dynamics of melt pools, vapor plume dynamics, and spatter generation.

Surface tension and evaporation both have a noticeable effect on the melt pool. Dai et al. [74] studied the process parameters of the thermal behavior, fluid dynamics, and surface morphology in a melt pool using a mesoscopic simulation model. The results indicated that the evolution of the melt pool was highly sensitive to the melt viscosity, surface tension, and recoil pressure during L-PBF. Bärtl et al. [75] investigated the ability of the aluminum alloy powder materials Al-Cr-Zr-Mn, Al-Cr-Sc-Zr, and Al-Mg-Sc-Mn-Zr to produce lightweight and high-performance structures by L-PBF. They regarded that both the surface tension and evaporation were potentially crucial factors dominating the melt dynamics, and the melt dynamics of materials with a lower surface tension and less evaporation were the most unstable. Table 4 summarizes the research on droplet spatter ejected from the “liquid” base of the melt pool.

### 3.3. Study of Powder Spatter Ejected from “Solid Base” of Substrate

Due to the entrainment effect of the gas flow, powder particles close to the laser zone of action are ejected and spattered. Ly et al. [64] performed an experimental comparison of the melt pool hydrodynamics of laser welding and L-PBF processes. In contrast to laser welding, the primary cause of spatter in L-PBF was not the laser-induced recoil pressure, but the entrainment effect of the ambient gas flow driven by the metal vapor on the micro-particles. The high-speed X-ray video imaging of the defects and melt pool performed by Leung et al. [76] supported the Ly et al. hypothesis about the generation of cold and hot entrainment spatter during L-PBF. Chen et al. [77] built a multi-phase flow model to investigate the spatter generation during L-PBF. The spatter phenomena were shown to be the result of metal vapor- and ambient gas-induced entrainment, which supported the findings of Ly et al. [64].

**Figure 12:**
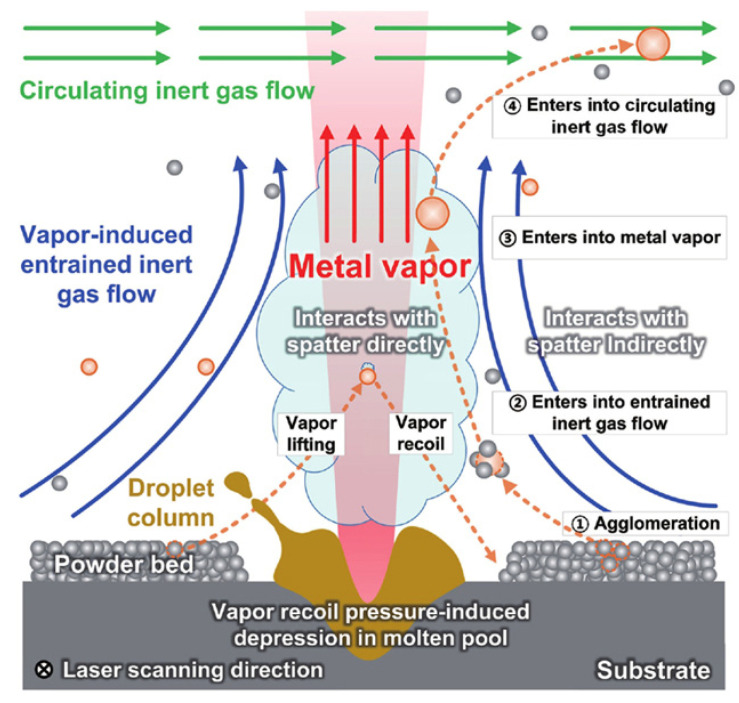
Schematic of spatter ejection process and interaction between vapor plume and spatter behavior in L-PBF. (Reprinted with permission from Ref. [61]. Copyright 2022 Chinese laser press.).

Gunenthiram et al. [78] used high-speed camera techniques to investigate the dynamic behavior of 316L stainless steel powder and 4047 aluminum–silicon alloy powder during the generation of spatter in L-PBF. As shown in Figure 12 [61], due to the heat transfer from the surrounding powder bed, the powder particles in close contact with the front and sides of the melt pool tended to agglomerate to form larger droplets. Some of the agglomerates were subject to an entrainment gas flow, which in turn were ejected as spatter. To establish the correlation between the scanning velocity and spatter generation, Zheng et al. [51] used a high-speed camera technique to investigate the effect of the scanning velocity on the generation and evolution of the metal vapor plumes during L-PBF of 304 stainless steel powder. The results indicated that the powder spatter generations are more closely related with the stability/evolution of the vapor plume and resulting melt-track, rather than the changing of the volumetric energy density (VED). The trend of an increasing number of spatters with an increasing VED was reported by Gunenthiram et al. [78]. The droplet spatter generated at the commencement of the scan trajectory was found to be the consequence of coupling between the melt pool and the inclined metal vapor plume. Table 5 summarizes the studies of the spatter from the solid substrate ejection.

### 3.4. Study of Spatter Generation Mechanism in Multi-Laser-PBF Fabrication Process

Recently, a multi-laser beam based on L-PBF has been applied to fulfil the growing demand for large-sized part manufacturing in aerospace and energy fields. Andani et al. [79] investigated the spatter behavior of Al-Si10-Mg powder during dual-beam L-PBF using a high-speed camera technique. They showed that the number of operating laser beams significantly influences the spatter creation mechanisms during the SLM process. A higher number of working laser beams induces a greater recoil pressure above the melting pools and ejects a larger amount of metallic material from the melt pools. However, there was no description of the interaction between the dual-beam laser and the material in the overlap region.

The mechanism by which a dual-beam laser generates spatter is distinct from that of a single-beam laser. Yin et al. [80] investigated the interaction between dual-beam lasers and the material in the overlap region during the dual-beam L-PBF of Inconel 718 alloy powder using a high-speed, high-resolution video imaging system. They proposed to use the spatter growth rate (*rs*) to quantitatively characterize the spatter behavior in multi-laser powder bed fusion (ML-PBF).

**Figure 13:**
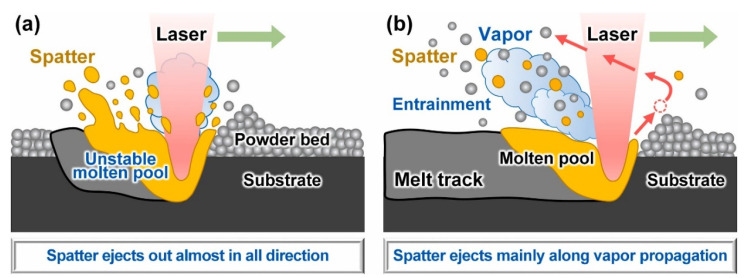
Schematic diagram of the transformation of the main mechanisms of spatter generation, which changes from (**a**) the vapor-induced recoil pressure with an almost homogeneous distribution of spatter ejection angle, into (**b**) the vapor-induced entrainment that majority of spatters eject along the direction of the metal vapor propagation. (Reprinted with permission from Ref. [80]. Copyright 2021 Elsevier B.V.).

According to experimental observations, Yin et al. [80] believe that most of the spatter in multi-laser L-PBF is due to metal vapor-induced entrainment (ejected from the “solid baes” of the substrate) rather than the metal vapor recoil pressure (ejected from the “liquid baes” of the melt pool). In fact, the *rs* in the vapor entrainment dominant stages is one order of magnitude higher than that in the unstable melt pool dominant stage disturbed by the recoil pressure and the collision of the two melt pools. This proves that the entrainment effect is dominant in the cause of the multi-laser-PBF spatter, as shown in Figure 13. A summary of the studies on the mechanism of the spatter generation during an ML-PBF process is shown in Table 6.

## 4. Disadvantage of Spatter

Spatter is an unpreventable by-product of the complex heat transfer process between the laser and the metal powder in L-PBF [20,30,54]. Spatter brings a negative influence to the process stability and the efficiency of the energy, which reduces the quality of the manufactured object and can potentially damage the machine [68]. In accordance with the current research, the disadvantages posed by spatter in L-PBF can be classified into three categories: (1) The effect of spatter on the printing processing: spatter can affect the powder re-coating in the next layer, and reduce the energy input efficiency of the laser and the operation stability of the powder re-coating device [63,81] as well as the optical lens. (2) The effect of spatter on structure and performance: spatter is not conducive to controlling the structure (e.g., voids, roughness) and performance (e.g., tensile properties, oxygen contents) of printed parts. (3) The effect of spatter on powder recycling: recycled powder can entrain spatter particles, resulting in a significant deterioration of powder quality. The use of recycled powder for forming parts can lead to a reduction in part performance.

### 4.1. Effect of Spatter on Printing Processing

According to the generation mechanism of spatter, it can be found that spatter has a negative influence on powder re-coating and energy absorption during L-PBF processing.

#### 4.1.1. Effect of Spatter on Powder Re-Coating

**Figure 14:**
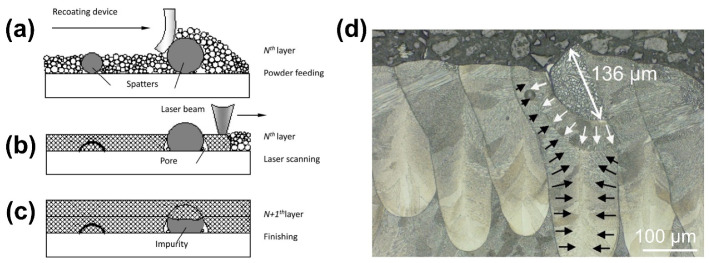
The effect of spatter on re-coating powder. (**a**) Pre-placing powders will be blocked when the next powder layer spreads; (**b**) spattering particles impede circulation of powders and cause voids nearby (before laser melting); (**c**) laser scanning can completely melt small spatter to achieve metallurgical bonding, while large spatter can only melt some of them. (Reprinted with permission from Ref. [63]. Copyright 2016 Elsevier B.V.). (**d**) A spatter particle of cross-section ~136 µm incites a solidification front (schematized with white arrows) that competes with the solidification fronts in the melt pool (schematized with black arrows). (Reprinted with permission from Ref. [82]. Copyright 2021 Elsevier B.V.).

Spatter particles that redeposit onto the powder bed hinder the powder re-coating, and voids between the spatter particles and powder can induce part defects. Figure 14 shows how spatter generated during L-PBF introduces voids and internal defects in the printed part. Wang et al. [63] discovered that the re-coating powders were influenced by the spatter particles due to a small amount of spatter attached to the surface of the printed parts during stacking, and the spatter particles caused the deformation of the scraper (Figure 14a). When the redeposited spatter particles are smaller than the layer thickness, after laser scanning, the spatter particles melted completely and were metallurgically bonded to the powder and the underlying part. If the redeposited spatter particles’ size exceeded the layer thickness, they did not melt completely, which induced voids between the powder and the spatter particles, as illustrated in Figure 14b. The voids remained after the scanning of the next layer, creating metallurgical defects, as illustrated in Figure 14c. Schwerz et al. [82] found the presence of spatter particles of approximately 136 µm in the cross-section of the part, illustrating how particles significantly larger than the nominal layer thickness were incorporated into the material despite recoating, and in the process, large spatter bumps of particles can cause damage to the scraper, as shown in Figure 14d.

**Figure 15:**
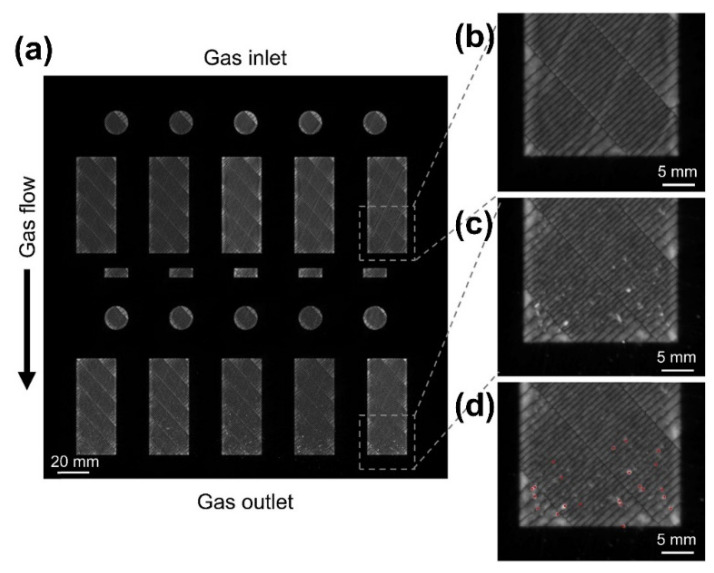
The results obtained by the monitoring system in conjunction with the spatter detection algorithm. (**a**) Sample long-exposure image consisting of the signals emitted during the exposure of a single layer on the entire build area. (**b**) A sample area near the gas inlet without any identified disturbances is highlighted for comparison. (**c**) Areas with disturbances are observed preferentially near the gas outlet. (**d**) A sample output from the spatter detection algorithm, in which the region shown in (**c**) is overlayed with detections. (Reprinted with permission from Ref. [82]. Copyright 2021 Elsevier B.V.).

In order to detect the distribution of the re-deposition of the spatters on the build area, a long-exposure near-infrared in situ monitoring associated with image analysis was employed to determine the exact locations using the EOS EOSTATE Exposure OT system [82]. This system consists of a 5-megapixel sCMOS (scientific complementary metal-oxide-semiconductor) camera positioned on top of the build chamber and comprises the entire build platform area in its field of view. A bandpass filter of 900 nm ± 12.5 nm is placed on the camera to filter the detection of the reflected laser to avoid the detection of the environmental noise. A sample image representative of a single layer can be observed in Figure 15a, samples near the gas inlet (Figure 15b) and gas outlet (Figure 15c) are shown separately. The long-exposure images revealed deviations in the form of high-intensity spots preferentially distributed towards the gas outlet, as in Figure 15c, the re-deposition spatter can be extracted by algorithms (Figure 15d). The spatter deposited near the gas outlet has been identified as one of the factors responsible for the rise of internal defects, which will be discussed in Section 4.2.

#### 4.1.2. Effect of Spatter on Energy Absorption

**Figure 16:**
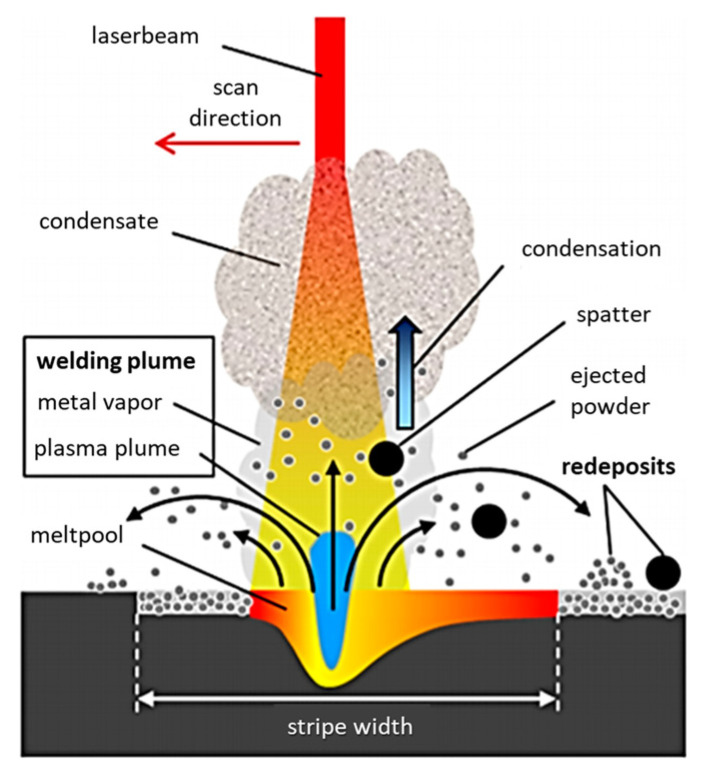
Spatter and other by-products pass through the laser stream and squander laser energy, condensate is the product of vaporized metal that quickly cools and condenses. (Reprinted with permission from Ref. [87]. Copyright 2016 Elsevier B.V.).

If spatter occurs in the laser path, it might result in an inefficient use of laser energy. Several studies have been done on the influence of spatter on the energy required to melt the powder. Ferrar et al. [83] first reported on the influence of gas flow on L-PBF in 2012. They demonstrated that by-products of processing in the laser path could absorb and scatter the laser beam, inducing laser beam attenuation and the generation of a lack of fusion. Anwar et al. [84] came to a similar conclusion in the selective laser melting of Al-Si10-Mg, implying that laser energy might be squandered on spatter, as shown in Figure 16. The laser beam irradiated the spatter particles that entered the beam path and consumed a significant amount of energy, which induced the incomplete melting of the powder and defects [85]. The accumulated spatter in the powder bed inevitably consumed the energy required to melt the fresh powder [86].

### 4.2. Effect of Spatter on Structure and Performance

**Figure 17:**
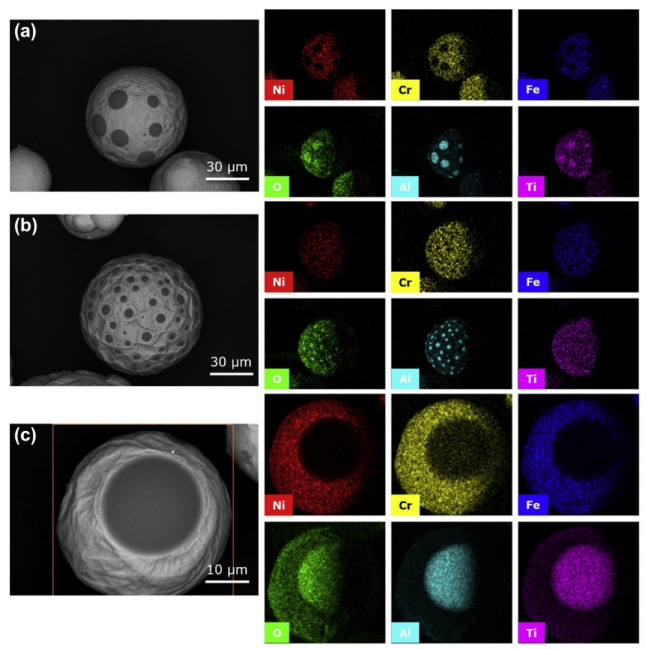
Back-scattered electron micrograph, and electron X-ray dispersive spectroscopy mapping of elements of Inconel 718 spatter collected from the ReaLizer SLM50. (**a**–**c**) shows that the dark spots mostly contain Al and O, and that the larger dark spots also contain Ti. EDS quantification results indicated that the oxides were a combination of Al_2_O_3_ and TiO_2_. (Reprinted with permission from Ref. [66]. Copyright 2018 Elsevier B.V.).

**Figure 18:**
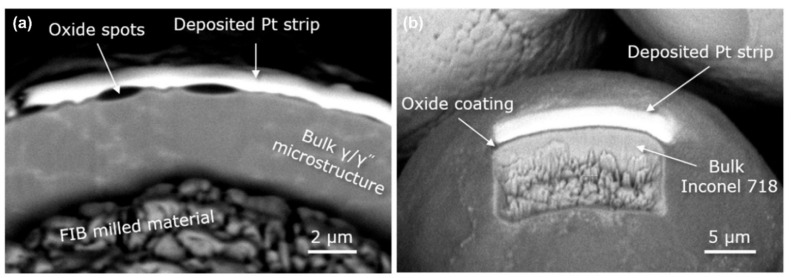
Back-scattered electron micrograph of particle with FIB sectioning to reveal microstructure and surface oxides (darker material) for particle (**a**) with oxide spots and (**b**) with oxide coating. The bright section is the sacrificial platinum strip deposited prior to ion milling. (Reprinted with permission from Ref. [66]. Copyright 2018 Elsevier B.V.).

Spatter causes a loss of laser energy, moreover, spatter re-deposition and oxidation also have an effect on the quality and structure of parts. A coating of oxide is generated on the spatter surface after L-PBF and the oxide layer greatly reduces the humidity of the liquid metal, which induces spheroidization [88,89]. The particles with an oxidized surface require more energy for melting and incorporation in the melt pool and in the bulk material, resulting in a lack of fusion [82]. The seriously oxidized spatter particles redeposit into the high-temperature melt pool, reversing the Marangoni convection flow direction [90,91]. Additionally, the oxidized spatter particles in the melt pool induce holes and defects [88,92]. The oxide composition of Inconel 718 spatter particles was evaluated by SEM-EDS by Gasper et al., as shown in Figure 17. In order to determine the extent of the oxidation of the spatter particles, a particle with oxide spots and fully oxidized particles were also analyzed by SEM-EDS with an in situ Focused Ion Beam (FIB), as shown in Figure 18.

**Figure 19:**
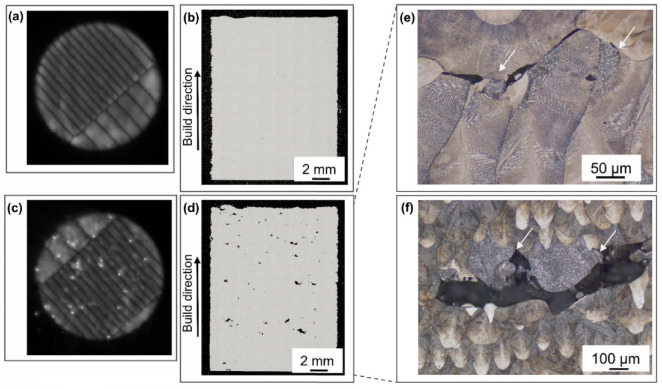
Cross section metallography of damaged testing [82]. (**a**) A low number of spatter redeposits are detected in specimens manufactured in the proximity of the gas inlet. (**b**) Metallographic analysis of these specimens reveals no major internal defects. (**c**) Detections of spatter redeposits can be abundant in specimens manufactured in the proximity of the gas outlet, (**d**) and these specimens present large internal defects. (**e**,**f**) are round particles with dendritic structure neighbor and lack of fusion defects, indicated by white arrows. (Reprinted with permission from Ref. [82]. Copyright 2021 Elsevier B.V.).

Schwerz et al. [82] investigated the effect of spatter on parts using destructive (metallographic analysis) and non-destructive (ultrasonic inspection) methods. It was discovered that the spatter redeposits zone included numerous internal defects. Based on the results of the redeposited spatters (Figure 19a,c), the cross-section metallography of samples with high and low rates of re-deposition spatters were analyzed. No obvious internal defects were found in the area with a low spatter re-deposition rate, as shown in Figure 19b. Numerous internal defects were found in the area with a high spatter deposition rate, as shown in Figure 19d. These internal defects are observed in conjunction with round particles with a dendritic structure, indicated by white arrows in Figure 19e,f, located with inter-melt pool boundaries, i.e., lack of fusion defects. Multiple internal defects larger than 500 µm were verified by the ultrasonic inspection as the layer thickness increased.

**Figure 20:**
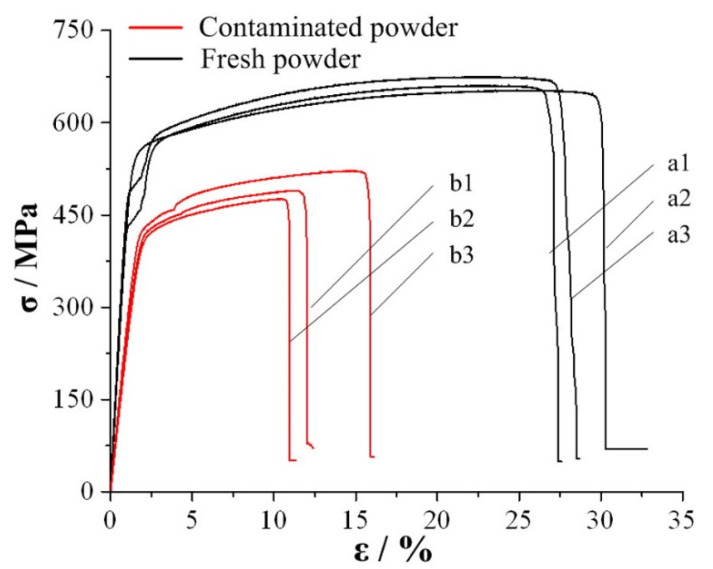
The stress–strain curves of tensile test pieces (fabricated from fresh and contaminated). (Reprinted with permission from Ref. [62]. Copyright 2015 Elsevier B.V.).

Spatter can cause a reduction in the tensile properties of the parts. Liu et al. [62] conducted tensile testing from fresh and contaminated 316L stainless steel powder, and the results showed that the mechanical properties of the specimens manufactured with contaminated powder are far inferior to those manufactured with fresh powder, as shown in Figure 20. Specimens with contaminated powder show considerably more voids in the fracture compared to specimens with fresh powder. These voids cause cracks and accelerate crack propagation during tensile testing, resulting in a dramatic reduction of mechanical properties in the specimens.

### 4.3. Effect of Spatter on Powder Recycling

Only 2 wt.% to 3 wt.% of the powder is selected for laser melting to metal pieces during L-PBF. Therefore, powder recycling is an efficient method of extending powder use [93]. However, recycled powder contains L-PBF by-products, which causes difficulties in powder recycling. Spatter particles are distributed in various sizes, a sieving mesh can easily remove most of the particles, but a small percentage of spatters smaller than the size of the original powders still remain. The powder recycling shows a distinct impact on the L-PBF process for powders of different components. (1) The 316L stainless steel powder is unique with an inherent SiO_2_ oxide layer on its surface that prevents the variable valence of metallic elements. It can be used up to 15 times in L-PBF without much affecting the mechanical properties of parts, but the oxygen content of the print increases with the number of recycles, and the part density decreases after 5 to 6 recycles [94]. (2) Ti-6Al-4V also contains an oxide layer on the surface; the elemental content of the powder remains nearly the same after 31 recycles, and the tensile strength, yield strength, and elongation are also almost unchanged [95]. (3) The recyclability of Al-Si10-Mg is poor, and its oxygen content doubles after 6 recycles [96]. (4) The steel alloy 17-4 PH showed a narrowing of the particle size distribution and a loss of tensile strength after 5 recycles [97]. (5) Hastelloy X is easy to be oxidized because it contains oxygenophilic elements such as Si, Cr, and Ni. Due to the wettability of Hastelloy X powder, it produces more spatters, which affects the re-cycling of the powder. He et al. [98] found that after 6 cycles of Hastelloy, the average particle size increased by 22% and the oxygen content increased by 48%, and the part porosity increased, resulting in a reduced part quality. The following Table 7 summarizes the number of re-cycle times available for different powders.

**Figure 21:**
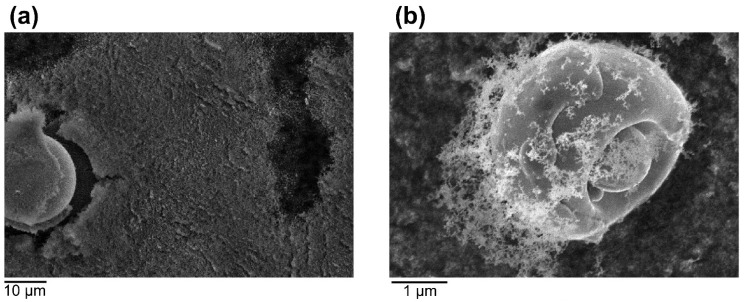
SEM images of condensate. (**a**) A heavy concentration of condensate. (**b**) Condensate on a captured laser spatter particle. (Reprinted with permission from Ref. [90]. Copyright 2019 Elsevier B.V.).

According to a study done by Marco Simonelli et al. [103], when powders are used for an extended period of time without sieving, numerous impurities mix with the powder and eventually become embedded in the surface of the manufactured part. Most of those impurities are spatter particles with the same composition as the slag produced during the conventional steel manufacturing process; the impurity consists primarily of SiO_2_ and other oxides, which can lead to impurity in the composition of the powder. Even after sieving, some spatter particles remain, and printing using powders containing spatter particles easily results in defects inside the part. Wang et al. [104] discovered that during L-PBF formation of a porous structure, the spatter particles in the recycled powder became inclusions in the part, influencing the part quality. Santecchia et al. [105] found that the environmental conditions in the build chamber can lead to the rapid condensation of vaporized material, and large amounts of condensate and spatter deposited together on the powder bed can affect the reuse of the powder. High concentrations of condensate and condensate on spatter particles were found by Sutton et al. [90] by SEM imaging, as shown in Figure 21.

The spatter has a negative effect on the whole process of L-PBF including the equipment (e.g., laser beam, scraper), current L-PBF manufacturing (e.g., structure and mechanical property), and subsequent L-PBF manufacturing (e.g., powder recycling). The generation of spatter will prevent the laser from directly irradiating on the powder bed, resulting in the loss of laser energy. The redeposited spatters will damage the scraper and become inclusions in the parts, which will reduce the structure and mechanical properties of the parts. Furthermore, spattering has an influence on the whole life cycle of powder. In current manufacturing, the spatters redeposit into the powder bed, and irregularly shaped spatter particles will become inclusions in the powder, increasing the powder’s oxygen concentration. These powders can result in inferior quality parts in subsequent manufacturing, leading to a decrease in the amount of powder recycling. Metal powders are more expensive than ingot metal, therefore, increasing the number of recycles of the powder is critical to making it more efficient to utilize. Spatter reduces powder quality and re-cycle times, and its removal can effectively improve powder usage efficiency, thus it is essential to research spatter countermeasures. The disadvantages of spatter are summarized in Table 8.

## 5. Spatter Countermeasures

The disadvantages of spatter include the equipment, components, and powders. Effective spatter countermeasures would extend equipment life, improve the parts’ quality, and enhance powder use. The full cycle of the spatter can be divided into three parts: generation, ejection, and re-deposition. In the generation stage, the generation of spatter can be suppressed by optimizing the laser volumetric energy density (VED), laser beam mode, and pressure of the building chamber. During the ejection and re-deposition stages, the protective gas flow is applied to remove the spatters which are in motion above the powder bed.

### 5.1. Process Parameters

In practice, regulating process parameters has emerged as a critical topic of study in reducing spatter effects during L-PBF. Process parameters such as (VED), scanning strategy, and build chamber pressures can affect the generation of spatter as follows: (1) Adopting a large spot combined with a low volume energy density can increase the depth of the melt pool and effectively suppress spatter. (2) The Bessel beam can be employed to stabilize the melt pool and reduce the generation of spatter. (3) The pre-sintering and re-coating printing strategy can reduce spatter generation. (4) Adding helium to the protective gas, reducing its oxygen content, and increasing the build chamber pressure can reduce spatter generation. A summary of studies on the control of L-PBF process parameters to reduce spatter generation during processing is shown in Table 9.

#### 5.1.1. Laser VED

**Figure 22:**
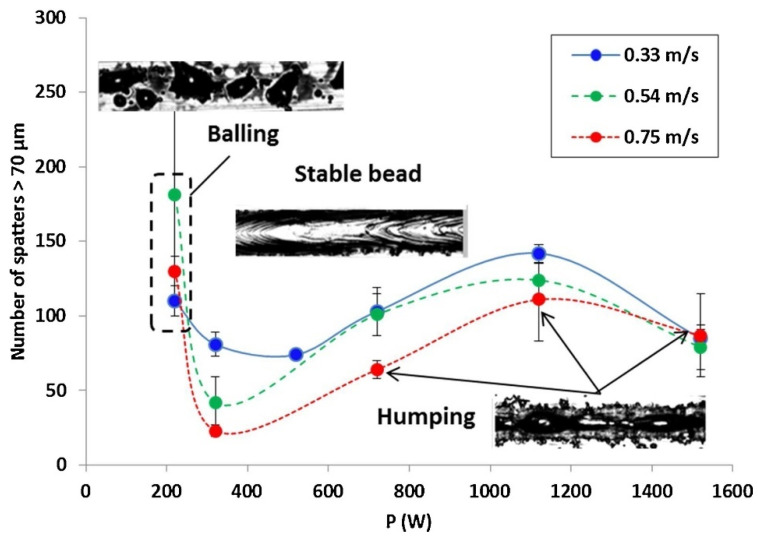
On a 316L stainless steel powder bed, the effect of laser power and scanning velocity on large-sized spatter, For low laser powers (*P* = 220 W) and resulting VED values severe balling occurs, that generates important spattering. The lower amount of spatters is obtained for *P* values just above the balling threshold (*P* = 320 W, *V* = 0.54 m/s and 0.75 m/s). (Reprinted with permission from Ref. [78]. Copyright 2018 Elsevier B.V.).

The laser VED affects the number and volume of spatters. The formula for calculating laser VED is $E_{V}=\frac{P}{Vd_{l}h_{p}}$. In the formula, P is the laser power, V is the scanning velocity, $d_{l}$ is the laser diameter, and $h_{p}$ is the layer thickness of the powder [127]. Gunenthiram et al. [78] demonstrated that the volume of spatter increased with increasing the VED, as seen in Figure 22. Mumtaz et al. [128] used pulse shaping techniques to precisely regulate the energy of the laser–material interaction zone, minimizing the generated spatter during L-PBF, which improved the top surface roughness of the parts and minimized the melt pool width. Shi et al. [115] demonstrated that by adjusting the energy density during single-layer formation, the spatter defects can be successfully reduced. The sample with the smoothest surface was produced when the linear energy density and the surface energy density was applied to 0.4 J/mm to 0.6 J/mm and 4 J/mm^2^ to 6 J/mm^2^, respectively.

- **Laser power:** The laser power applied affects the number and volume of spatters, in most situations, studies have shown that the higher the laser power input, the more severe the spatter behavior. Andani et al. [52] concluded that decreasing the laser power would reduce spatter in L-PBF, and the laser power dominates the effect on spatter generation. Chen et al. [46] demonstrated that adjusting the power intensity and distribution of the laser beam to maintain the melt pool temperature between the melting and boiling points can significantly reduce spatter generation.
- **Scanning velocity:** The velocity of the laser scanning will affect the generation of spatter. Andani et al. [52] considered that increasing the laser scanning velocity would reduce spatter in L-PBF. Gunenthiram et al. [78] studied the number of spatters at different scanning velocities (V = 0.33~0.75 m/s) and found that the higher the scanning velocity, the less the number of hot spatters, as shown in Figure 22. However, a high scanning velocity leads to a longer scanning path, which increases the cold spatter caused by entrainment.
- **Laser diameter:** The laser spot size during L-PBF can significantly affect the melt dynamics and droplet spatter generation [117]. There are two reasons for the variation of the spot size: passive variation and active variation. For passive changes, the lens could be deformed due to thermal expansion and contraction induced by the incident high-energy laser, so that the spot size varies during laser conduction. The active variation is to adjust the spot size of the laser artificially. Gunenthiram et al. [78] demonstrated a possible way to entirely suppress the spatter by using a large spot when the melt pool is sufficiently deep. Sow et al. [116] investigated the influence of a large laser spot on L-PBF and concluded that combining a large spot with a low VED significantly improved the L-PBF in terms of the process stability, spatter reduction, and component density.
- **Layer thickness:** A high layer thickness results in a large amount of spatter. Schwerz et al. conducted experiments with layer thicknesses of 80 µm, 120 µm, and 150 µm, and found that the number of redeposited spatters increased with the layer thickness [82]. The heat of the melt pool cannot be conducted quickly by the surrounding powder as the layer thickness rises, which leads to the instability of the melt pool, and the number of spatters increases accordingly. However, due to the limited area of laser irradiation, the increase in the spatter will slow down when the layer thickness reaches a certain thickness. Zhang et al. [38] found that spatter generation slows down when the layer thickness exceeds twice the size of the powder particles.

#### 5.1.2. Laser Mode

The generation of spatter is influenced by the mode of the laser beam used in L-PBF. The main modes of lasers currently used in L-PBF are: the Gaussian beam, inverse Gaussian (annular) beams, flat-top beam, and Bessel beam. Several studies have shown that Bessel beams are significantly better than Gaussian beams in L-PBF.

**Figure 23:**
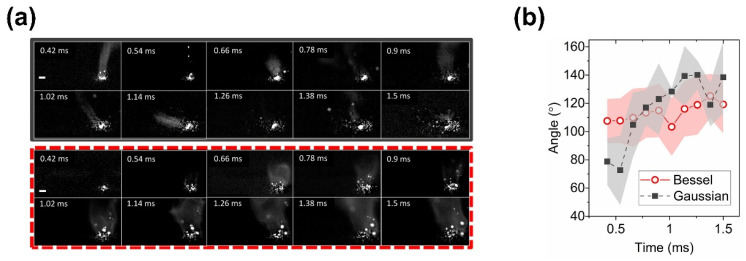
(**a**) Side views of melt pool captured using Gaussian (top, gray border) and Bessel (bottom, dashed red border) lasers; (**b**) angle of melt pool steam relative to horizontal level as a function of time. (Reprinted with permission from Ref. [129]. Copyright 2021 AAAS).

- **Gaussian beam:** Less spatter would be produced while printing with L-PBF equipment that uses Bessel beams. The Gaussian beam produces more spatter and the spatter is ejected at a higher velocity, this is due to the higher recoil forces generated by the Gaussian-like thermal distribution of the laser beam on the melt pool [129].
- **Inverse Gaussian (annular) beams:** Compared to the Gaussian beam, the inverse Gaussian (annular) can reduce the creation of spatter and increase the geometric tolerance of the 3D parts [119].
- **Flat-top beam:** L-PBF with a flat-top beam generates less and slower spatter than Gaussian beam and inverse Gaussian (annular) beams, as stated by Okunkova et al. [119].
- **Bessel beam:** The Bessel beam helps stabilize the melt pool to reduce spatter. Nguyen et al. [118] investigated the possibility of using Bessel beams for ultrafast laser processing in AM, indicating that Bessel beams might alleviate the negative impacts of spatter in L-PBF. Tumkur et al. [129], utilizing high-speed imaging to detect the dynamics of melt pool, found that Bessel beams stabilize the melt pool’s turbulence, increase their solidification times, and reduce spatter generation (Figure 23).

#### 5.1.3. Printing Strategy

The scanning strategy can be divided into two categories: scanning path and pre-sintering method. The checkerboard scanning path can reduce the generation of spatter, and when the scanning direction is consistent with the gas flow direction, the spatter can be effectively removed. Pre-sintering with a low-energy density can also effectively suppress the generation of spatter.

- **Generation of spatter:** Rivalta et al. [130] found that the hexagonal (outside-in verse) scanning strategy would produce more spatter. It is speculated that when hexagonal patterns are used for component manufacturing, the time between adjacent scan tracks rises, the temperature range becomes too wide, so more energy is required to heat the surrounding environment, resulting in increased spatter. A checkerboard scan approach can help to reduce the generation of spatter.
- **Removal of spatter:** The trajectory of the spatter is dependent on the direction of the laser scan. The movement trajectory of most spatters is opposite to the scanning direction. The spatter can be effectively removed if the direction of the spatter movement is consistent with the protective gas flow. However, the gas flow direction is determined by the design of the equipment, and the optimizing of the laser scanning direction can be performed. Effective spatter removal can be achieved by changing the direction of the laser scanning so that the trajectory of the spatter is consistent with the direction of the protecting gas flow. Anwar et al. [84] found that spatters re-depositioned near the outlet of the build chamber were greatly decreased when the laser scans were against the direction of the protective gas flow, but large particle spatters were still difficult to be removed [85,120].

Pre-sintering can form necks between powder particles, which is often used in electron powder bed fusion (E-PBF) to prevent powder redistribution. Similarly, pre-sintering can be introduced into L-PBF to reduce the generation of spatters. Metal powder has a significantly higher thermal absorption rate than solid bulk metal, the amount of spatter generated during L-PBF can be reduced by using a scanning strategy of a low-energy-density laser pre-sintering [103]. Khairallah et al. [69] demonstrated that combining high laser power with pre-sintering can significantly suppress spatter generation, particularly oversized (~200 mm) back-ejected spatter (spatter in the backward direction) at the start of the scanning trajectory. Achee et al. [131] used pre-sintering to prevent spatter and denudation, and they found that the control of spatter and denudation was most effective when the pre-sintered VED was 1–4 J/mm^3^. Moreover, Annovazzi et al. [132] indicated that pre-sintering powder could help prevent spattering. Constantin et al. [133] demonstrated that adding a re-coating step can increase the part quality compared to the conventional L-PBF process.

#### 5.1.4. Pressure of Build Chamber

**Figure 24:**
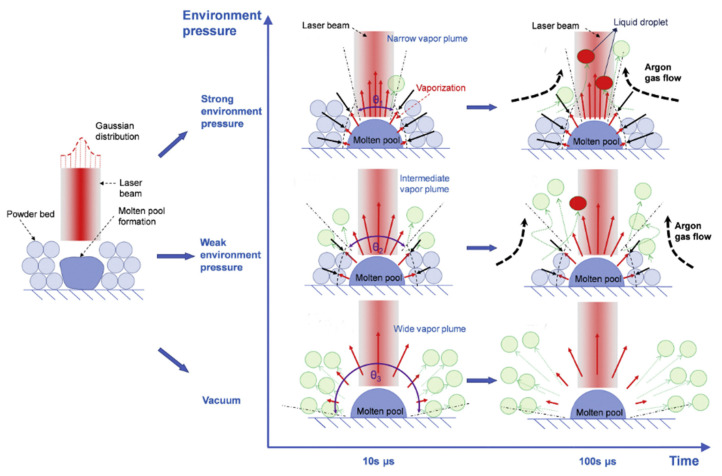
Schematic of powder spattering behavior as a function of time and environment pressure during L-PBF.(Reprinted with permission from Ref. [36]. Copyright 2018 Elsevier B.V.).

The environmental pressure within the build chamber affects spatter generation. As the environmental pressure increased, the total amount of spatter dropped gradually, but the hot spatter generated by argon gas flow entrainment increased [36], and the smoothness and continuity of the built layers was degraded [35], as illustrated in Figure 24**.** Kaserer et al. [122] investigated the effect of pressure variation on L-PBF. They discovered that the amount of spatter produced by the pure titanium and maraging steel 1.2709 used in the study did not change considerably when the process pressure was varied between 200 mbar and atmospheric pressure.

Based on research of the laser–powder bed interaction at sub-atmospheric pressures, Bidare et al. [121] demonstrated that while the ambient pressure decreased as gas entrainment rose, the expanding laser plume prevented the powder particles from reaching the melt pool. Li et al. [123] investigated the flow of gas, the gas–solid interaction, and the powder behavior in L-PBF at various ambient pressures. It was noted that as ambient pressure decreased, powder spatter particle and divergence angles increased, which is consistent with the Guo et al. [36] experiment results. They considered that as the ambient pressure decreased, the number of spatters grew monotonically. Spatter movement was suppressed by increasing the ambient pressure during L-PBF. Annovazzi et al. [132] demonstrated that vacuum conditions and a high laser velocity are detrimental to the stability of the powder layer, which induced more spatter.

#### 5.1.5. Protective Gas

The influence of inert gas on spatter is due to two factors: the primary component of the gas (Ar, He, N_2_, 50% Ar–50% He mixture) and the secondary component of the gas (O). The inert gas’ protective effect is due to its major component. Helium, which has a positive influence on spatter suppression, has a high thermal conductivity (ten times that of Argon). As a result of this high thermal conductivity, the temperature of the melt pool is lower and the back punch is smaller, resulting in less spatter generated. However, the rarity of Helium is the reason for its high price, in the range of about 3 to 6 times per cylinder compared to argon, so, taking this into account, there is more use of argon gas for production. Oxygen, being a tiny component of the inert gas, can cause spatter to increase and oxidize; therefore, lowering the oxygen level in the inert gas helps to suppress spatter generation.

**Figure 25:**
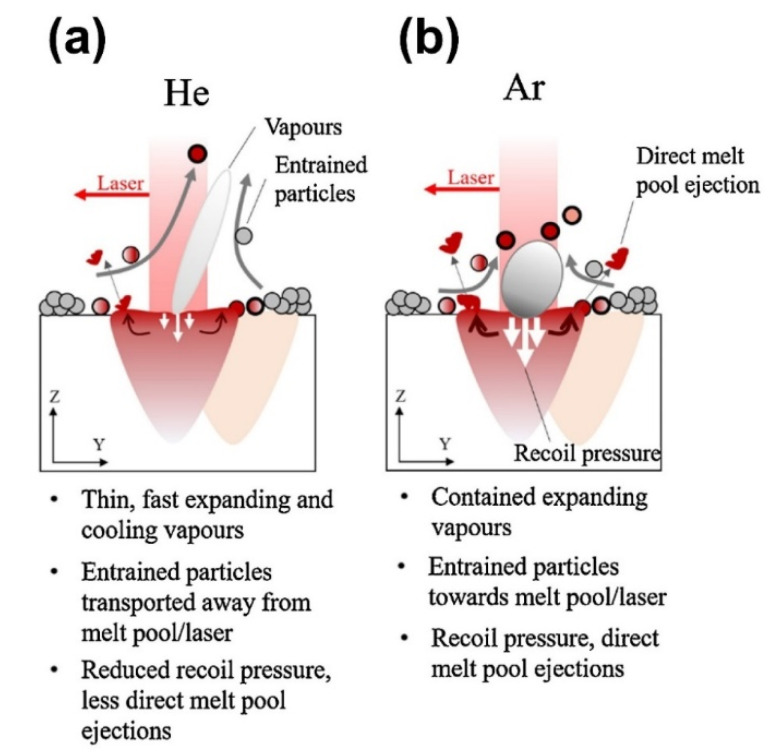
The interaction of the laser–powder bed with (a) Helium and (b) Argon in the L-PBF protective gas. (Reprinted with permission from Ref. [125]. Copyright 2021 CIRP.).

- **Primary components of inert gases:** Pauzon et al. [125] studied the effect of protective gas on L-PBF of Ti-6Al-4V powder in three different conditions: pure argon, pure helium, and a helium and argon mix (oxygen content was controlled at 100 ppm). In comparison to the common use of argon, studies have indicated that using pure helium or a mixture of helium and argon can reduce hot spatter by at least 60% and ~30%, respectively, as shown in Figure 25. No influence of different protective gases on the number of cold spatters was detected. The study also found that adding helium to the gas can help cool spatter more quickly, which is important for limiting powder-bed degradation throughout L-PBF.
- **Secondary component of the inert gas:** According to Wu et al. [124], the oxygen concentration in the protective environment increased considerably, resulting in the generation of spatter and an increase in the oxygen content of spatter during flight. By decreasing the oxygen level of the build chamber, the spatter generation can be reduced.

Reducing the oxygen content in the build chamber is an efficient approach to prevent spatters from generating. Through multiple gas circulations, the equipment can decrease the oxygen level in the build chamber as much as feasible. Furthermore, keeping the build chamber at slightly above the atmospheric pressure can prevent the entry of oxygen from outside the equipment and, at the same time, the flowing inert gas can eliminate the generated spatter.

#### 5.1.6. Gas Flow Strategies

Most modern L-PBF equipment using gas flow removes process by-products from the process zone to enable an undisturbed process. Ladewig et al. [87] examined the influence of the protective gas flow uniformity and rate on single-laser tracks and the hatching process during the building procedure of bulk material. The efficiency of spatter removal decreased as the velocity of the protective gas flow reduced. Chien et al. [134] proposed to optimize and calibrate the inert purge airflow in an L-PBF build chamber using simulation framework methods such as coupled computational fluid dynamics (CFD) and the discrete element method (DEM). Wang et al. [126] created a full-scale geometric model to explore the interaction between the protective gas flow and the laser-induced spatter particles. The flow field was found to be steady up to a height of 30 mm above the surface of the powder bed. It was discovered that printing in this region could improve the final quality due to the consistent high-velocity flow of the protective airflow in the center of the powder bed, which removed by-products such as spatter.

### 5.2. Equipment and Materials for L-PBF

In addition to regulating process parameters, research on L-PBF equipment and materials has become a major focus for mitigating the effect of spatter. These two research areas will also contribute to the future commercialization of L-PBF technology. A summary of the research on L-PBF equipment and materials is shown in Table 10.

#### 5.2.1. Research on L-PBF Equipment

**Figure 26:**
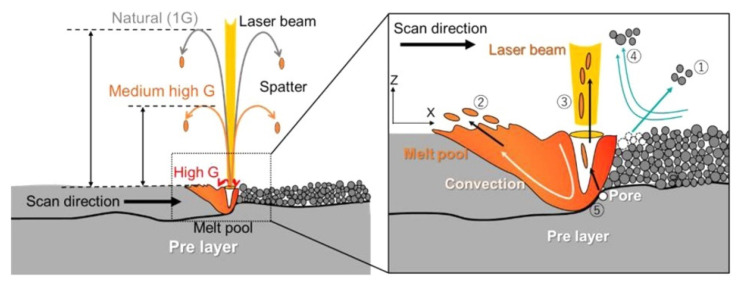
Classification and suppression of spatter under high gravitational acceleration: ① solid spatter; ② metallic ejected spatter; ③ powder agglomeration spatter; ④ entrainment melting spatter; and ⑤ defect induced spatter. (Reprinted with permission from Ref. [139]. Copyright 2021 Elsevier B.V.).

Spatter generation can be reduced by optimizing L-PBF equipment. Koike et al. [138,139] developed a high-gravity L-PBF system that generated a strong gravitational field by centrifugal acceleration. At a high gravity acceleration of more than 10 G, the spatters were greatly suppressed. As illustrated in Figure 26, the height of the spatter trajectory was inversely related to the increased gravitational acceleration. They noted that when a suitably strong gravitational acceleration was applied, spatter generation was dramatically suppressed.

Philo et al. [135] used numerical simulations to investigate the interaction between the gas flow and spatter. They discovered that the parameters of the protective gas inlet and outlet in the build chamber (e.g., the radius of the inlet nozzles, the heights of the inlet and outlet) significantly affect the flow velocity, uniformity, and spatter concentration. Xiao et al. [136] simulated the flow field in an L-PBF build chamber to optimize the flow-field structure. The flow-field state was evaluated using the particle tracer method. It was shown that the flow-field distribution was made more uniform by structural optimization, which can improve the ability of the gas flow to entrain spatter.

**Figure 27:**
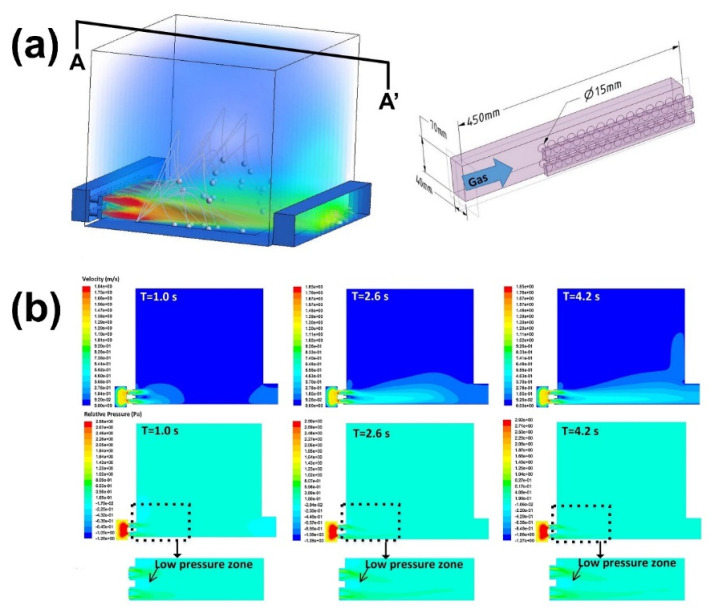
CAD model of counter-Coanda effect in L-PBF build chamber: (**a**) the counter-Coanda effect design that employs another row of nozzles directly under the primary nozzles; (**b**) A-A’ plane: transient velocity and pressure field. (Reprinted with permission from Ref. [137]. Copyright 2020 Elsevier B.V.).

To increase the capability for spatter removal, Zhang et al. [137] proposed a novel design for the gas flow system in the build chamber, as illustrated in Figure 27. The effect of the gas flow on the solid particles was obtained using the fully coupled CFD-DPM fluid–particle interaction model. The new design increased the spatter removal rate by reducing the Coanda effect, which substantially affected the spatter removal process. In addition, another row of nozzles was added directly under the primary inlet nozzles.

Current novel L-PBF machines generally use multi-laser beams to print simultaneously to increase efficiency, which generates more spatter. Optimizing the equipment, especially the build chamber, to remove spatter has become a major concern for many L-PBF machine manufacturers. SLM Solutions GmbH (Lubeck, Germany) has introduced adopting the building chamber to a high pressure in order to minimize the spatter activity, which hence has lowered spatter generation [144]. Through the streamlined special-shaped design of the flow channel, Bright Co. Ltd. (Xi’an, China) [145] reduced the vortex current at the outlet of the protective gas, and the steam plume and spatters are ensured to be blown away and not redeposit on the forming surface during forming, which solves the quality problem of the forming surface during printing. General Electric Co. invented a gas flow system for an additive manufacturing machine that uses a gas flow parallel to the powder bed to remove by-products (including spatter) from the L-PBF manufacturing process [146]. The MYSINT 100 3D printer from SISMA [147], Italy, has a stable and uniform flow field to ensure spatter removal efficiency.

#### 5.2.2. Research on Powder Material

The physical properties and oxygen content of the powder can also contribute to differences in spatter behaviors, which can be reduced by a high viscosity, high thermal conductivity, and high density. Powders with a low oxygen content caused significantly less spatter in L-PBF.

- **Physical properties:** High thermal conductivity and densification have a positive effect on spatter suppression. Due to the higher thermal conductivity of aluminum in the liquid state 316L, the laser energy can be rapidly dissipated into the substrate, limiting the vaporization of the aluminum alloy and the resulting spattering [78]. Gunenthiram et al. [78] pointed out that due to the densification effect, the melt pool will be located below the surface of the powder bed, which will inhibit the generation of spatter. The melt pools formed by the laser irradiation of different powder particles have varying viscosities which influence the generation of spatter. Leung et al. [140] investigated the laser–material interaction of 316L stainless steel powder and 13–93 bioactive glass powder during L-PBF at short time scales. The results indicate that droplet spatters are easily generated in a low-viscosity melt (e.g., 316L) because of the strong Marangoni-driven flow. By contrast, a high-viscosity melt (e.g., 13–93 bioactive glass) reduces spatter generation by dampening the Marangoni-driven flow.
- **Oxygen content:** For the raw powder used in L-PBF, the higher the oxygen content, the greater the melt pool instability and the greater the probability of spattering. Fedina et al. [143] found that with the oxygen content of the powder rose, the number of spatters increased, whereas the other chemical elements remained relatively constant. They suggested that the increase in oxygen might have affected the powder spattering. Additionally, an increase in the powder oxygen content led to an increase in the oxygen content of the melt pool, which in turn affected the flow behavior of the fluid in the melt pool, leading to spattering as the melt pool broke into molten droplets [148]. Fedina et al. [142] investigated L-PBF dynamics and powder behavior by comparing water-atomized and gas-atomized powders. They discovered that the water-atomized powder had more frequent spatter ejection and speculated that the higher oxygen level in the powder caused the melt pool to become unstable, resulting in an excessive number of spatters.

Manufacturers are also concentrating their efforts on developing powder materials suitable for L-PBF, offering a wide variety of powder materials such as various titanium alloys, nickel alloys, aluminum alloys, and cobalt–chromium alloy powder materials for the aerospace, automotive, and biomedical fields.

## 6. Conclusions

This paper reviews the literature on the in situ detection, generation, effects, and countermeasures against spatter in L-PBF. The main points of this review are summarized as the following:

(1) **In situ detection system for spatter during L-PBF:** The detection methods are based on the physical properties (trajectory and brightness) of the spatter and melt pool. The variances in the trajectory and brightness lead to differences in the sensors and light sources of the detection system.
  - **Sensor:** Due to the complex and unpredictable trajectories of the spatters in the 3D space compared to the melt pool, detection requires multiple sensors and sophisticated algorithms. A 3D detection solution with a quadruple-eye sensor combined with algorithms has been applied in a visible-light detection system. The emergence of 3D detection solutions provides more information in three dimensions, which improves the accuracy of the spatter detection.
  - **Light source:** Compared to the bright high-temperature melt pool, the spatters consist of both bright hot droplet spatters and dark cold powder spatters. The motion of dark cold powder spatter can hardly be captured without an external light source. Therefore, a visible light source must be applied to enable the detecting of two types of spatters.
(2) **Mechanism of spatter generation in L-PBF:** spatter can be divided into droplet spatter from the “liquid base” of the melt pool and powder spatter from the “solid base” of the substrate.
  - **Droplet spatter from the “Liquid base” of the melt pool:** The droplet spatter originates from the instability of the melt pool. The Marangoni effect and the metal vapor recoil pressure generated on the surface of the melt pool lead to the spatter ejection from “liquid base” of the melt pool.
  - **Powder spatter from the “Solid base” of the substrate:** Powder spatter is induced by the entrainment effect of the ambient gas flow driven by the metal vapor. A low-pressure area is generated near the high-speed moving metal vapor, and the surrounding inert protective gas will be “entrained” to the vicinity of the melt pool, driving the powder spatter to be ejected from the “solid base” of the substrate.
(3) **Spatter effects during L-PBF:** Spatter has negative effects not only on the equipment and quality of parts, but also on the whole life cycle of the powder. Therefore, spatter significantly affects both the current L-PBF manufacturing and the subsequent L-PBF manufacturing.
  - **Equipment:** the laser light path will be obstructed by the ejected spatter, and the scraper will be damaged by the redeposited spatter.
  - **Current L-PBF manufacturing:** redeposited spatter can cause deterioration in the part structure and mechanical property.
  - **Subsequent L-PBF manufacturing:** the spatters redeposit into the powder bed to be inclusions, resulting in a decrease in the quality of the re-cycle powder and affecting the subsequent L-PBF manufacturing.
(4) **Countermeasures for spatter in L-PBF:** for the full cycle of spatter (generation–ejection–redeposition), the countermeasures for spatter are divided into spatter generation suppression and spatter removal.
  - **Spatter generation suppression:** the generation of spatter can be suppressed by optimizing the laser volumetric energy density (e.g., raising the scanning velocity, lowering the laser power, decreasing the layer thickness, and increasing the laser spot), laser beam mode (Bessel beams), and pressure of the building chamber.
  - **Spatter removal efficiency:** The gas flow removes process by-products from the process zone to enable an undisturbed process. Simulation framework methods (CFD and DEM) and a full-scale geometric model are employed to optimize the flow filed structure. A high-velocity gas flow under a certain value (counter-Coanda effect) applied in the center of the powder bed greatly improves the efficiency of spatter removal.

## 7. Future Research Directions

As the main technology in metal AM, L-PBF is evolving toward a greater efficiency, precision, speed, and fabrication of large-sized parts. However, spattering has caused negative influence on the product quality during L-PBF. The following trends characterize the directions of research on L-PBF spatter behavior:

(1) **Study of spatter behavior under multiple lasers:** Multi-laser synergy has been the main solution to achieve more efficient fabrication of large-sized parts. However, the mechanism of spatter becomes more complicated due to the enhancement of metal vapor, the Marangoni effect, and entrainment under the multi-laser interaction. Additionally, each laser induces both “liquid-based” and “solid-based” ejected spatters, and the amount of spatter increases dramatically using multiple lasers. The spatter is more difficult to be removed by gas flow due to the large-scaled build chamber. Therefore, the research of spatter in multi-beam manufacturing has become more urgent.
(2) **Improving the quality of in situ spatter detection:** The combination of a visible-light high-speed camera and X-ray imaging technology in spatter detection coincides with the development trend of spatter detection [149]. The combination of the two methods enables us to study spatter behaviors from the inside (melt pool) to the outside (powder bed), and gain more information on the behaviors of the spatter. The multi-sensor system is indispensable in the research of spatter and the number of sensors can be expanded based on the existing quadruple-eye sensor.
(3) **Information processing using artificial intelligence:** The data volume of the multi-sensor system could exponentially increase with the addition of data sources such as temperature, radiant intensity, light intensity information, acoustic signals, and images of melt pools and spatters. Therefore, machine learning (supervised, semi-supervised, unsupervised) is necessary for the efficient processing of the multi-source and heterogeneous data.
(4) **Countermeasures for spatter:** At present, simulations are commonly used to study the countermeasures of spatter, and the raw data used in the simulations come from their detection. Improving the comprehensiveness and accuracy of detection information is conducive to the actual application of the simulation of spatter countermeasures.
(5) **Commercial L-PBF equipment:** Several companies (e.g., Concept laser, EOS, SLM solutions) have developed systems for detecting melt pools during L-PBF manufacturing, but there is still a lack of spatter detection in the equipment. As a result of the complex spatter behaviors and serious negative impact in L-PBF, it is necessary to remove as much of the spatter as possible by using dynamical control of the protective gas flow field. The addition of an in situ spatter detection system enables the dynamical feedback of the control of the gas flow field.

## Figures and Tables

**Table 1:** Characteristics obtained from different in situ detection techniques.

In Situ Detection Technology	Obtained Characteristics
Visible-light high-speed camera	Surface characteristics
X-ray video imaging	Internal structure Flow behavior of melt inside the melt pool
Infrared video imaging	Temperature distribution Flow behavior of gas
Schlieren video imaging	Gas flow propagation and distribution

**Table 2:** In situ detection system for L-PBF.

System	Sensors	Spatial Resolution (µm/Pixel)	Temporal Resolution (Hz)	Light Source	Object of Detection	Materials	References
Coaxial	Phantom V2512 by Vision Research Inc.	14.6	23,077 (Max. 1,000,000)	—	Hot spatter	316L	Zhang et al. (2022) [38]
Three-dimensional off-axis	FPS1000 by The Slow Motion Camera Company	18–24	1000	—	Spatter and ejecta	—	Barrett et al. (2018) [43]
	Phantom v1210 by Vision Research Inc.	40	60,000	CAVILUX HF	Spatter	316L	Eschner et al. (2019) [44]
	Asler aca640–750 μm USB3	200	750	—	Spatter	316L	Eschner et al. (2022) [45]
Two-dimensional off-axis without light source	Photron Fastcam Mini AX200	—	5000	—	Denudation and vapor plume	316L	Chen et al. (2022) [46]
	I-SPEED high-speed CMOS camera	—	50,000		Plume	Ti-6Al-4V	Zheng et al. (2021) [47]
	Qianyanlang 5KF10	14	9800		Spatter and powder	316L	Wang et al. (2021) [48]
	Pco. dimaX HS4	11	3000		Spatter	316L	Yang et al. (2020) [49]
	—	11.7	2000		Melt pool and spatter	316L	Zhang et al. (2019) [50]
	I-SPEED 716	—	20,000		Vapor plume and spatter	304	Zheng et al. (2018) [51]
	Fastcam 1024 PCI	—	6000		Plume and spatter	Al-Si10-Mg	Andani et al. (2018) [52]
	FASTCAM Mini UX50/100	—	5000		Plume and spatter	304 L	Ye et al. (2018) [53]
Two-dimensional off-axis with light source	Phantom V2012 by Vision Research Inc.	3.92–5.70	100,000	CAVILUX^®^ pulsed high-power diode laser light source	Droplet and melt pool	Inconel 718	Yin et al. (2020) [34]
	Phantom V1212 by Vision Research Inc.	—	37,500	Diode laser	Ejecta	Inconel625	Nasser et al. (2019) [54]
	Phantom V2512 by Vision Research Inc.	1.5–11	8000	Lumencor SOLA SM white light source	Spatter and denudation	316L	Biadre et al. (2018) [35]
X-ray	Argonne National Laboratory, USA	—	50,000	—	Melt pool and spatter	Ti-6Al-4V	Zhao et al. (2017) [28]
		1	54,310		Powder spatter	316L/Al-Si10-Mg	Guo et al. (2018) [36]
		2	400,000		Keyhole *	Ti-6Al-4V	Cunningham et al. (2019) [55]
		—	45,259–135,776		Spatter	Al-Si10-Mg/Ti-6Al-4V	Young et al. (2020) [56]
	55 keV monochromatic X-rays	6.6	5100		Melt pool	Invar 36	Leung et al. (2019) [57]

* Keyhole: also known as the depression zone, is wrapped by the gas–liquid interface and penetrates through the melt pool.

**Table 3:** Summary of spatter classification studies.

**Classification According to the “In-Process Analysis”**			
**Classification Principle**	**Materials**	**Spatter Categories**	**References**
Vapor recoil pressure, Marangoni effect	316L, CoCr; 316L, Ti-6Al-4V; Al-Si10-Mg, Ti-6Al-4V	Metallic ejected spatter	Liu et al. (2015) [62]; Wang et al. (2017) [63]; Ly et al. (2017) [64] Young et al. (2020) [56]
Vapor recoil pressure	316L; Al-Si10-Mg, Ti-6Al-4V	Powder spatter	Liu et al. (2015) [62]; Young et al. (2020) [56]
Entrainment effect	316L, Ti-6Al-4V; Al-Si10-Mg, Ti-6Al-4V	Powder spatter; Entrainment melting spatter	Ly et al. (2017) [64]; Young et al. (2020) [56]
Instability during laser–pore interaction	Al-Si-10Mg, Ti-6Al-4V	Defect-induced spatter	Young et al. (2020) [56]
Agglomeration	Al-Si-10Mg, Ti-6Al-4V	Agglomeration spatter	Young et al. (2020) [56]
**Classification According to the Post-Mortem Analysis**			
**Classification Principle**	**Materials**	**Spatter Categories**	**References**
Appearance and Composition	Inconel 718	(i) Particles similar to virgin gas-atomized particles; (ii) Particles with morphology different to gas-atomized; (iii) Larger singular particles with different morphologies; (iv) Particles with oxide spots; (v) Particles covered with oxide; (vi) Small particles; (vii) agglomerates	Gasper et al. (2018) [66]
	Al-Si10-Mg	Hollow droplets, semi-hollow droplets, solid droplets	Yang et al. (2020) [67]

**Table 4:** Summary of research on droplet spatter ejected from the “liquid base” of the melt pool.

Generation Mechanism	Materials	References
Surface tension	Ti-6Al-4V, TiC	Dai et al. (2020) [74]
	Al-Cr-Zr-Mn, Al-Cr-Sc-Zr & Al-Mg-Sc-Mn-Zr	Bärtl et al. (2022) [75]
Vapor recoil pressure	316L	Khairallah et al. (2016) [68]
	Inconel 718	Yin et al. (2019) [41]
		Yin et al. (2020) [34]
Explosion	Ti-6Al-4V	Zhao et al. (2019) [71]
	Cu-10Zn	Yin et al. (2021) [72]
Laser energy uneven deposition	316, Ti-6Al-4V	Khairallah et al. (2020) [69]
Movement process of melt and powder	316L	Wang et al.(2021) [48]

**Table 5:** A summary of the studies on spatter from solid substrate ejection.

Generation Mechanism	Material	References
Metal vapor-induced entrainment	316L, Ti-6Al-4V	Ly et al. (2017) [64]
	316L, 4047 aluminum–silicon	Gunenthiram et al. (2018) [78]
	316L	Chen et al. (2020) [77]
	GH4169	Yin et al. (2022) [61]
Metal vapor recoil pressure	304	Zheng et al. (2018) [51]

**Table 6:** A summary of the studies on the mechanism of spatter generation during ML-PBF process.

Dominant Mechanism	Material	Research Content	References
Vapor-induced recoil pressure	Al-Si10-Mg	Number of laser beams ↑, Recoil pressure ↑, Number of spatters ↑.	Andani et al. (2017) [79]
Vapor-entrainment effect	Inconel 718	Spatter growth rate (*rs*) in vapor entrainment dominant stages is one order of magnitude higher than that in unstable melt pool dominant stage	Yin et al. (2021) [80]

**Table 7:** Summary of the re-cycle times available for different powders.

Material	Powder Parameters	Re-Cycle Times	References
316L	20~45µm	10–15	Gorji et al. (2019) [99] Delacroix et al. (2022) [94]
Ti-6Al-4V	<63 µm	21–31	Tang et al. (2015) [100] Quintana et al. (2018) [95]
Al-Si10-Mg	20~63 µm	6–30	Cordova et al. (2019) [96] Mohd et al. (2020) [101]
17-4 PH	15~45 µm	5–11	Nezhadfar et al. (2018) [97] Jacob et al. (2017) [102]
Hastelloy X	20~60 µm	6	He et al. (2022) [98]

**Table 8:** Summary of studies on the disadvantages of spatter.

Disadvantage		Material	References
Printing processing	Laser energy loss	316L	Liu et al. (2015) [62]
		Ti-6Al-4V	Pal et al. (2020) [106]
	Abrasion of scraper	CoCr	Wang et al. [63]
		Hastelloy X	Schwerz et al. [82]
Structure and mechanical property (current L-PBF manufacturing)	Spatter oxidation (oxygen content of part increases due to redeposited spatters)	316L	Hatami et al. (2021) [107]
		Al-Si10-Mg	Lutter et al. (2018) [108]
	Lack of fusion	CoCrMo	Darvish et al. (2016) [109]
		Al-Si10-Mg; Ti-6Al-4V	Young et al. (2020) [56]
		Ti-6Al-4V	Pal et al. (2020) [106]
		316L	Obeidi et al. (2020) [110]
		Inconel 718	Ladewig et al. (2016) [87]
		CoCr	Wang et al. (2017) [63]
	Increase in surface roughness	17-4 PH	Ali et al. (2019) [111]
		Hastelloy-X	Esmaeilizadeh et al. (2019) [112]
Powder recycling (subsequent L-PBF manufacturing)	Porosity increase	Ti-6Al-4V	Strondl et al. (2015) [113]
	Mixing of spatter particles	Al-Si10-Mg	Lutter et al. (2018) [108]
		304 L	Obeidi et al. (2020) [110]
	High oxygen content (oxidized spatter in recycled powder increases)	Hastelloy X	Esmaeilizadeh et al. (2019) [112]
		316L	Lu et al. (2022) [114]

**Table 9:** Summary of studies on the regulation of process parameters.

Process Parameters	Spatter Countermeasures	Materials	References
Laser VED	Decrease laser power	316L, TC4	Liu et al. (2015) [62] Shi et al. (2021) [115] Luo et al. (2021) [42] Chen et al. (2022) [46]
	Increase laser scanning velocity	Al-Si10-Mg	Andani et al. (2018) [52]
	Increase laser spot	316L, 4047 Al-Si alloy; Inconel 625; Ti-6Al-4V	Gunenthiram et al. (2018) [78] Sow et al. (2020) [116] Young et al. (2022) [117]
	Reduce layer thickness	316L	Zhang et al. (2022) [38]
Laser beam modes	Bessel beams	316L	Nguyen et al. (2021) [118]
	Flat-top beam	Co-Cr	Okunkova et al. (2014) [119]
Printing Strategy	Pre-sintering	316L, Al-Si10-Mg, Ti-6Al-4V	Simonelli et al. (2015) [103]
		Ti-6Al-4V, 316L	Khairallah et al. (2020) [69]
	Scan in the opposite direction to the gas flow	Al-Si10-Mg	Andani et al. (2017) [79] Anwar et al. (2018) [85] Anwar et al. (2019) [120]
Ambient pressure	Increasing the ambient pressure	316L	Bidare et al. (2018) [121]
		Pure (CP) titanium grade 2, Maraging steel 1.2709	Kaserer et al. (2020) [122]
		316L	Guo et al. (2018) [36] Li et al. (2021) [123]
Protective Gas	Reducing the oxygen content of atmosphere	316L	Wu et al. (2016) [124]
	Increase gas flow velocity (without blowing away the powder bed)	Inconel 718	Ladewig et al. (2016) [87]
	Adding helium to protective gas	Ti-6Al-4V	Pauzon et al. (2021) [125]
	Printing in the central area of the powder bed	Ti-6Al-4V	Wang et al. (2021) [126]

**Table 10:** A summary of the research on L-PBF equipment and materials.

	Materials	Spatter Countermeasures	References
L-PBF equipment	316L, Aluminum	Uniformity of flow field	Philo et al. (2018) [135] Xiao et al. (2021) [136]
	316L	Prevent powder from blowing away	Zhang et al. (2020) [137]
	316L	High gravity powder bed	Koike et al. (2021) [138,139]
Powder materials	316L, 13-93 bioactive glass	Increasing the viscosity of melt	Leung et al. (2018) [140]
	AISI 4130; 316L	Reducing the oxygen content of powder	Heiden et al. (2019) [141] Fedina et al. (2020) [142] Fedina et al. (2021) [143]
